# Synthesis of Sulfonyl Chlorides from Aryldiazonium Salts Mediated by a Heterogeneous Potassium Poly(heptazine imide) Photocatalyst

**DOI:** 10.1021/acsorginorgau.1c00038

**Published:** 2021-12-13

**Authors:** Yevheniia Markushyna, Markus Antonietti, Aleksandr Savateev

**Affiliations:** Department of Colloid Chemistry, Max-Planck Institute of Colloids and Interfaces, Am Mühlenberg 1, 14476 Potsdam, Germany

**Keywords:** carbon nitride, photocatalysis, organic synthesis, sulfonyl chloride, heterogeneous catalysis

## Abstract

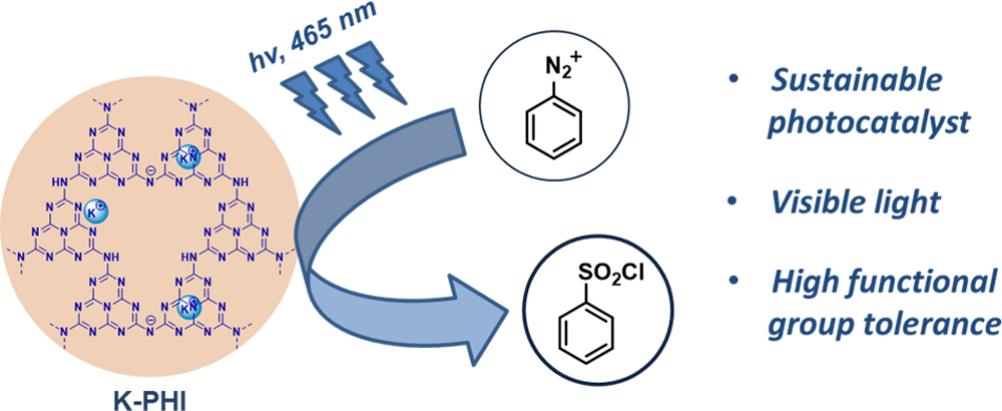

Visible light photocatalysis is a tool in synthetic chemistry that allows us to utilize the energy of photons via photoinduced electron transfer to promote diverse organic reactions. Herein, a heterogeneous transition metal-free material, a type of carbon nitride photocatalyst, potassium poly(heptazine imide), is employed to produce sulfonyl chlorides from arenediazonium salts under mild conditions (visible light irradiation, room temperature) with 50–95% yields. The method is suitable for the synthesis of both electron rich and electron deficient compounds, and it shows high tolerance toward different functional groups (halides, ester, nitro, cyano groups). Thus, a sustainable photocatalytic alternative to the Meerwein chlorosulfonylation reaction is offered.

## Introduction

**Figure 1:**
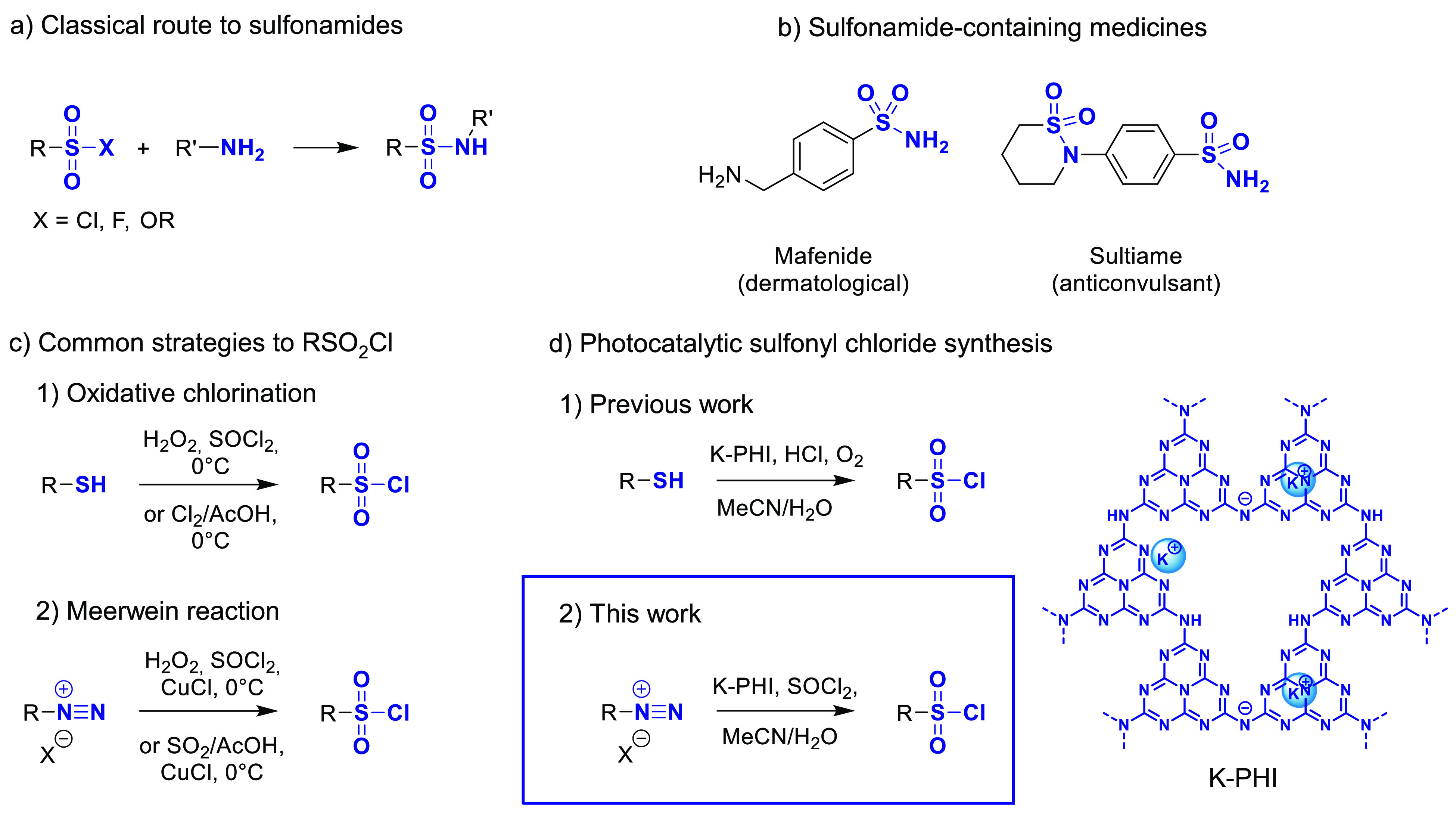
(a) Classical route to sulfonyl amides. (b) Medicines containing sulfonamide linkage. (c) Common strategies for sulfonyl chlorides synthesis. (d) Photocatalytic methods for sulfonyl chlorides synthesis.

Synthesis of sulfonyl chlorides is one of the most important procedures and is part of the daily routine of an organic chemist. These compounds are the main precursors in the synthesis of sulfonyl amides, at both the laboratory and industrial scale (Figure 1a). Sulfonamides, in turn, represent one of the biggest class of biologically active compounds used in pharmaceutical, agrochemical, materials, and food industries (Figure 1b).^1−3^ Apart from this, sulfonyl chlorides also serve as key intermediates in the synthesis of sulfonyl fluorides, sulfonate esters, sulfones, and sulfinic acids.^4^ In general, synthesis of sulfonyl derivatives from amines or alcohols belongs to the most commonly used reactions in pharmaceutical research.^5^ In addition, sulfonyl chlorides are also used in functional group protection or activation of unreactive sites.^6^ As a source of alkyl and aryl radicals, they also find application in photocatalytic coupling reactions.^7^

A common method of sulfonyl chlorides synthesis is a Sandmeyer-type reaction proposed by Meerwein et al. using copper salts, such as CuCl or CuCl_2_, as a catalyst for single electron transfer (SET) (Figure 1c).^8^ In the original work, arenediazonium salts, obtained from amines, reacted with SO_2_ in aqueous medium to yield the product in low-to-moderate yields. The method was further improved by using a concentrated solution of SO_2_ (30%) in glacial acetic acid under ice bath cooling (temperatures below 5 °C) to increase the yields.^9^ The mixture resulted in two phases anyway due to the poor solubility of diazonium salts and sulfonyl chlorides in aqueous system. Furthermore, the presence of water is not favorable for the reaction, as it leads to the hydrolysis of sulfonyl chlorides to sulfonic acids. Apart from the targeted product, there are also several side products possible, such as the Sandmeyer products chloroarene, disulfide, and sulfone (Scheme S1). Therefore, improvement of existing methods for synthesis of sulfonyl chlorides is necessary.

Visible light photocatalysis is seen as one of the prominent alternatives to conventional chemistry, which through harvesting solar light and followed by photoinduced electron transfer enables many important chemical transformations, including H_2_ production,^10−13^ CO_2_ conversion,^14−16^ pollutant degradation,^17,18^ and also organic synthesis.^19,20^ Previously, the photocatalytic alternative to the Meerwein method was proposed by Jacobi von Wangelin et al. in 2017.^21^ To enable this reaction, the authors used a transition-metal-based catalyst tris(2,2′-bipyridine)ruthenium(II)dichloride [Ru(bpy)_3_]Cl_2_. However, due to low abundance of platinum group metals, the costs of Ru-complexes are high. On the contrary, sulfonyl chlorides are rather bulk commodities. Therefore, cheap and recyclable photocatalysts for synthesis of this class of organic compounds are highly desirable.

The family of carbon nitrides, polymer materials built of mainly C and N atoms, are organic semiconductors and a popular choice in photocatalysis due to low cost of precursors (several EUR per gram, if synthesis performed on the lab-scale) and high performance.^19,22,23^ In addition, the heterogeneous nature of the catalyst allows for easy separation from the reaction mixture by filtration or centrifugation and its further reuse. Such a combination of features of these materials is expected to improve the sustainability of organic synthesis. Originally, carbon nitrides were developed for the use in sustainable energy processes, such as water splitting for hydrogen production; however, in the past decade, they have been more and more investigated as catalysts in organic synthesis as an alternative to the established transition-metal-based photocatalysts.^22^ In particular, potassium poly(heptazine imide) (K-PHI), a crystalline type of carbon nitride, has shown high performance in organic photocatalysis, such as reduction,^24,25^ oxidation reactions,^26,27^ or redox neutral reactions.^28^ Due to the valence band (VB) position located at +2.36 V vs NHE, K-PHI is able, via photoinduced electron transfer, to oxidize amines^26^ and alcohols^27^ and even perform the thermodynamically challenging oxidation of halide anions.^29^ Poly(heptazine imide) and carbon nitrides, in general, are used as supports for single atoms.^30−33^ Thus, Fe-PHI performs exceptionally well in the thermodynamically challenging oxygenation of hydrocarbons under illumination with visible light.^34^ K-PHI also forms stable radical anions, which can be practically applied, for example, in oxygen sensing.^35,36^

Recently, we have already shown that sulfonyl chlorides can be obtained by the photocatalytic oxidative chlorination of aromatic thiols and arylthioacetates (Figure 1d(1)).^37^ Nevertheless, this method is not universal due to the limited availability of the starting materials as a result of their challenging synthesis. In addition, oxidative chlorination typically has limited tolerance to the functional groups due to the harsh reaction media. Therefore, other strategies for the synthesis of sulfonyl chlorides, especially those using photocatalysis, remain in demand.

In this work, we develop a method of sulfonyl chloride synthesis from in situ generated SO_2_ and HCl, and arenedizonium tetrafluoroborates, which are prepared from the corresponding aromatic amines via diazotation with nitrous acid. The reaction is mediated by heterogeneous potassium poly(heptazine imide) photocatalyst.

## Results and Discussion

Diazonium salts were prepared according to the reported procedure.^38^ We used SO_2_ and HCl that are formed in situ upon hydrolysis of SOCl_2_ with an equimolar amount of water. Like in many photocatalytic organic reactions catalyzed by carbon nitrides, acetonitrile was chosen as a solvent due to its ability to disperse the catalyst and dissolve all molecular components of the reaction mixture: arenediazonium salts, SO_2_, HCl, and sulfonyl chlorides. This allows all components except the catalyst to be in one phase and mitigate all previously described limitations.^9^ For the optimization of reaction conditions on a 0.025 mmol scale of 4-bromophenyldiazonim tetrafluoroborate **1a**, we started with 20 equiv of SOCl_2_ and H_2_O (Table 1).

**Table 1:**
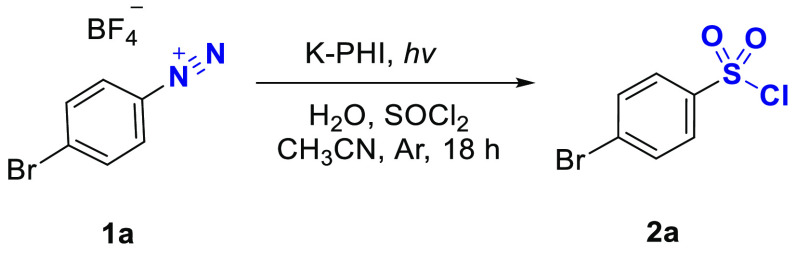
Reaction Condition Optimization

entry	substrate, mmol	light, nm	SOCl_2_ and H_2_O equiv vs **1a**	yield (conversion), %^h^
1^a^	0.025	465	20	85 (100)
2^a^	0.025	white	20	85 (100)
3^a^	0.050	white	20	85 (100)
4^a^	0.100	white	20	85 (100)
**5**^b^	**0.025**	**465**	**10**	**95 (100)**
6^c^	0.025		10	0
7^d^	0.025		10	0
8^e^	0.025	465	10	0
9^f^	0.025	465	10	60
10^g^	0.025	465	10	0

a Conditions: K-PHI (4 mg); SOCl_2_ (37.0 μL, 0.5 mmol); H_2_O (10 μL, 0.56 mmol); MeCN (1 mL); *T* = 25 °C; atmosphere = Ar; 18 h.

b Conditions: K-PHI (4 mg); SOCl_2_ (18.5 μL, 0.25 mmol); H_2_O (5 μL, 0.28 mmol); MeCN (1 mL); *T* = 25 °C; atmosphere = Ar; 18 h.

c The same as footnote *b* but without catalyst and light.

d The same as footnote *b* but without light.

e The same as footnote *b* but without catalyst.

f The same as footnote *b* but without adding water explicitly.

g The same as footnote *b* but using anhydrous acetonitrile. Reaction mixture was prepared in the glovebox.

h Conversion and yield obtained from ^1^H NMR analysis of the reaction mixture.

Illumination of the reaction mixture with 465 nm photons led to complete conversion of the substrate and formation of the sulfonyl chloride **2a** with 85% yield (entry 1). A similar yield of **2a** was obtained under white light (entries 2–4). The main byproduct in the reactions listed in entries 1–4 was *p*-bromochlorobenzene. Upon decreasing the excess of thionyl chloride to 10 equiv, we improved selectivity and obtained **2a** with 95% yield (entry 5). The reaction did not proceed in the absence of light and/or the photocatalyst (entries 6–8). The experiment without addition water resulted in 60% yield due to minor contents of water in the solvent as the latter was used without any additional treatment (entry 9). By means of Karl Fischer titration, the water content in acetonitrile was measured to be 640 ppm, which is translated into 0.036 mmol of H_2_O, and therefore sufficient to produce amount of SO_2_ required by the stoichiometry of the reaction (Table S1). To confirm the role of water in acetonitrile, we used acetonitrile with water contents <252 ppm. The reaction did not give **2a** (entry 10). These results clearly confirm the necessity of water for the reaction to proceed.

The performance of other carbon nitride materials and some transition metal-based complexes was evaluated in the reaction of 4-chlorophenyldiazonim tetrafluoroborate **1f** (Table 2, entries 1–6). The commonly employed in photocatalysis material graphitic carbon nitride, g-CN, gave **2f** with only 15% yield. A carbon nitride material with an enhanced surface area, mesoporous graphitic carbon nitride (mpg-CN),^39^ gave **2f** with 98% yield. Taking into account the comparable yield of **2f** in the case of K-PHI and mpg-CN, but with a more complex preparation procedure of the latter that requires a hard template, the use of K-PHI is advantageous. Another type of PHI-based catalyst, Na-PHI, gave **2f** with 12% yield, possibly due to limited absorption in the visible range.^40^ The Ir- and Ru-based complexes gave **2f** with 99% yield in this reaction. However, the use of precious metals is undesirable. Thus, the K-PHI photocatalyst is the most reasonable choice for catalyzing this reaction. Additionally, recycling experiments were performed with K-PHI catalyst, which unambiguously confirmed its stability and high performance over several rounds of use (Table 2, entries 1, 7, 8). The experiment with DABCO-Bis(sulfur dioxide) (DABSO) as a source of SO_2_ did not yield sulfonyl chloride **2f** but mainly diarylsulfone (Table 2, entry 9; Figure S2).

**Table 2:**
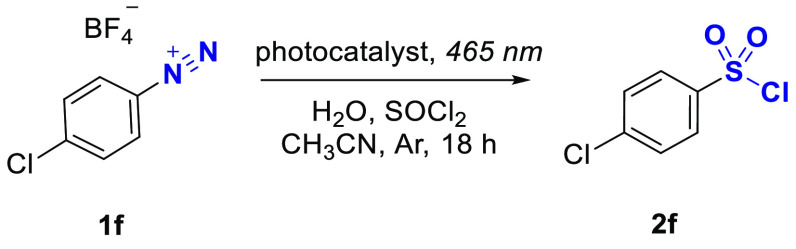
Catalyst Scope Investigation^a^

entry	catalyst	light, nm	SOCl_2_ and H_2_O equiv vs **1f**	yield (conversion), %^e^
**1**	**K-PHI**	**465**	**10**	**99 (100)**
2	g-CN	465	10	15 (15)
3	mpg-CN	465	10	98 (100)
4	Na-PHI	465	10	12 (13)
5	Ru(bpy)_3_Cl_2_	465	10	99 (100)
6	Ir(ppy)_3_	465	10	99 (100)
7^[^b^]^	K-PHI 2nd run	465	10	98 (100)
8^[^c^]^	K-PHI 3rd run	465	10	97 (100)
9^[^d^]^	K-PHI	465	10	0 (100)

a Conditions: catalyst (4 mg); substrate (0.025 mmol); SOCl_2_ (18.5 μL, 0.25 mmol); H_2_O (5 μL, 0.28 mmol); MeCN (1 mL); *T* = 25 °C; atmosphere = Ar; 18 h.

b Second run of the recycled K-PHI.

c Third run of the recycled K-PHI.

d Reaction with DABSO (0.125 mmol) and 2 M HCl in Et_2_O (62 μL, 0.125 mmol).

e Conversion and yield obtained from ^1^H NMR analysis of the reaction mixture.

**Scheme 1:**
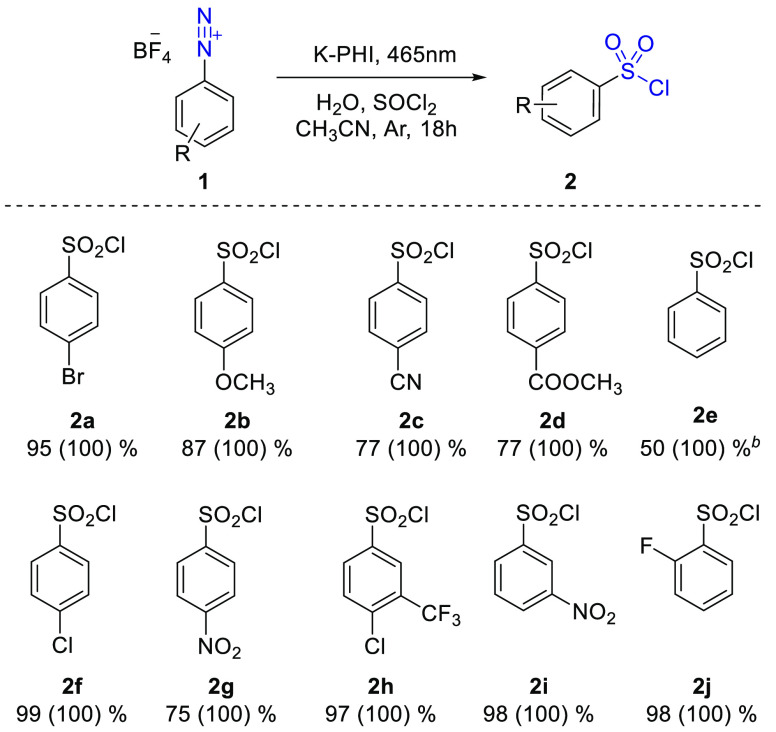
Scope of Substrates Used in the Chlorosulfonylation Reaction Catalyzed by K-PHI Reaction conditions: **1** (0.025 mmol); K-PHI (4 mg); SOCl_2_ (18.5 μL, 0.25 mmol); H_2_O (5 μL, 0.28 mmol); MeCN (1 mL); *T* = 25 °C; atmosphere = Ar; irradiation with 465 nm LED; 18 h. Dichloromethane was used as a solvent. The yield and conversion (in parentheses) are given in percent.

A scope of arenediazonium salts with various substituents at *o*-/*m*-/*p*-positions **1a**–**j** were studied under the optimized conditions. Except for 2-methoxy-substituted phenyldiazonium salt, both electron rich and electron poor arylsulfonyl chlorides **2a**–**j** were obtained with 50–95% yields (Scheme 1). This is beneficial as compared to the conventional method, which yields only electron-deficient and electron-neutral sulfonyl chlorides in good yields.^8,9^ Also, the proposed method shows high tolerance toward various functional groups, such as halogen, ester, nitro, and cyano groups. This serves as additional proof of this photocatalytic approach being milder than the established chemical process, which shows a lower compatibility with functional groups.

It is worth mentioning that special conditions should be used for synthesis of unsubstituted sulfonyl chloride **2e** as it reacts with acetonitrile and forms acetanilide, which was detected by ^1^H NMR (Supplementary Note 1 in the Supporting Information). Thus, the reaction with **1e** was performed in dichloromethane to avoid this problem.

**Figure 2:**
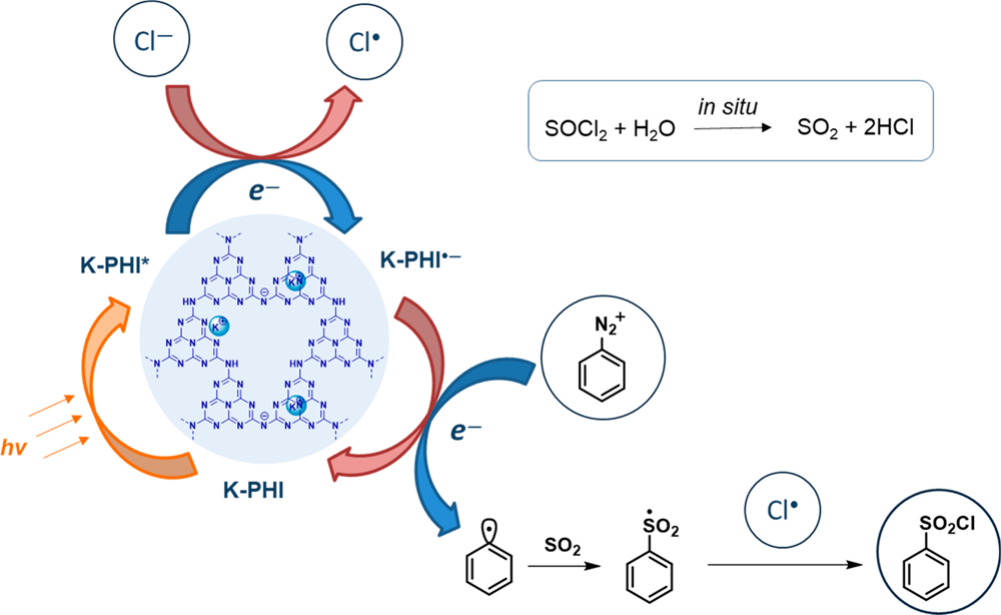
Proposed mechanism of the synthesis of sulfonyl chlorides from arenediazonium salts catalyzed by K-PHI.

Based on our experience and previous studies,^21^ the following mechanism was proposed (Figure 2). First, upon reaction with water, thionyl chloride provides the reaction mixture with SO_2_ and hydrochloric acid. In the photocatalytic cycle, under irradiation with a 465 nm LED, K-PHI forms an exited state K-PHI*, which can undergo the redox half-reactions with active species present in the reaction mixture. On the VB site, K-PHI as a strong oxidant (VB position at 2.36 V vs NHE) is able to oxidize chlorine anion (Cl^—^) (oxidation potential 1.36 V vs NHE), as was shown in our previous studies.^29^ This leads to the formation of a K-PHI radical-anion (K-PHI^•—^) and chlorine radical (Cl^•^). On the CB site, by the analogy with Cu(I) salts in the Meerwein reaction, arenediazonium salts undergo a single electron reduction by K-PHI^•—^. Upon release of the nitrogen molecule, it forms an aryl radical, which simultaneously reacts with the SO_2_ molecule to form an S-centered sulfonyl radical. The reduction potential of benzenediazonium tetrafluoroborate is 0.08 V vs NHE,^41^ while the CB of K-PHI is located at −0.35 V vs NHE and thus allows for this reductive half-reaction. In addition, SET to the diazonium salt followed by reduction to the phenyl radical was proved by the radical trapping experiment with DMPO by GC-MS (Figure S4). In the final step, the phenylsulfonyl radical reacts with the chlorine radical and yields the desired product–arenesulfonyl chloride.

## Conclusions

In this work, we presented the photocatalytic method of sulfonyl chloride synthesis from arenediazonium salts catalyzed by a heterogeneous catalyst, which is composed of only lightweight C, N, K elements and from this perspective is more sustainable. Potassium poly(heptazine imide) (K-PHI), a member of the carbon nitride family, affords sulfonyl chlorides bearing both electron donating and electron withdrawing groups with high yields. Necessary SO_2_ and Cl^–^ amounts are conveniently obtained by the reaction of SOCl_2_ and water in situ. The reaction was carried out under mild conditions, under visible light irradiation, and at room temperature, which all gave a high functional group tolerance. Thus, a convenient method toward organic sulfonyl chlorides with a heterogeneous, inexpensive catalyst was developed.

## Supporting Information Available

Materials and characterization methods, synthesis procedures, supplementary notes, characterization of compounds (PDF)

## References

[ref1] Feng M.; Tang B.; Liang S. H.; Jiang X. Sulfur Containing Scaffolds in Drugs: Synthesis and Application in Medicinal Chemistry. Curr. Top. Med. Chem. 2016, 16 (11), 1200–16. 10.2174/1568026615666150915111741. 26369815 PMC4877035

[ref2] Devendar P.; Yang G. F. Sulfur-Containing Agrochemicals. Top. Curr. Chem. 2017, 375 (6), 82 10.1007/s41061-017-0169-9. 28993992

[ref3] McGorrin R. J. The Significance of Volatile Sulfur Compounds in Food Flavors. In Volatile Sulfur Compounds in Food; American Chemical Society: 2011; Vol. 1068, pp 3–31.

[ref4] Tanaka K. Sulfonic Acids, Esters, Amides and Halides as Synthons. In Sulphonic Acids, Esters and their Derivatives, Patai S., Rappoport Z., Eds.; Wiley: New York, 1991; pp 401–452.

[ref5] Roughley S. D.; Jordan A. M. The medicinal chemist’s toolbox: an analysis of reactions used in the pursuit of drug candidates. J. Med. Chem. 2011, 54 (10), 3451–79. 10.1021/jm200187y. 21504168

[ref6] Avery M. A. Protective Groups in Organic Synthesis. Third Edition By Theodora W. Greene and Peter G. M. Wuts. John Wiley & Sons, New York. 1999. xxi + 779 pp. 16 × 24 cm. ISBN 0-471-16019-9. $84.95.. J. Med. Chem. 1999, 42 (25), 5285 10.1021/jm990518h.

[ref7] Chaudhary R.; Natarajan P. Visible Light Photoredox Activation of Sulfonyl Chlorides: Applications in Organic Synthesis. ChemistrySelect 2017, 2 (22), 6458–6479. 10.1002/slct.201701156.

[ref8] Meerwein H.; Dittmar G.; Göllner R.; Hafner K.; Mensch F.; Steinfort O. Untersuchungen über aromatische Diazoverbindungen, II. Verfahren zur Herstellung Aromatischer Sulfonsäurechloride, Eine Neue Modifikation der Sandmeyerschen Reaktion. Chem. Ber. 1957, 90 (6), 841–852. 10.1002/cber.19570900602.

[ref9] Gilbert E. E. Recent Developments in Preparative Sulfonation and Sulfation. Synthesis 1969, 1969 (1), 3–10. 10.1055/s-1969-34188.

[ref10] Chen X.; Shen S.; Guo L.; Mao S. S. Semiconductor-based photocatalytic hydrogen generation. Chem. Rev. 2010, 110 (11), 6503–70. 10.1021/cr1001645. 21062099

[ref11] Ahmad H.; Kamarudin S. K.; Minggu L. J.; Kassim M. Hydrogen from photo-catalytic water splitting process: A review. Renewable Sustainable Energy Rev. 2015, 43, 599–610. 10.1016/j.rser.2014.10.101.

[ref12] Zhao C.; Chen Z.; Shi R.; Yang X.; Zhang T. Recent Advances in Conjugated Polymers for Visible-Light-Driven Water Splitting. Adv. Mater. 2020, 32 (28), 1907296 10.1002/adma.201907296. 32483883

[ref13] Chen Z.; Wang H.; Xu J.; Liu J. Surface Engineering of Carbon Nitride Electrode by Molecular Cobalt Species and Their Photoelectrochemical Application. Chem. - Asian J. 2018, 13 (12), 1539–1543. 10.1002/asia.201800487. 29696798

[ref14] Wang S.; Han X.; Zhang Y.; Tian N.; Ma T.; Huang H. Inside-and-Out Semiconductor Engineering for CO2 Photoreduction: From Recent Advances to New Trends. Small Structures 2021, 2 (1), 2000061 10.1002/sstr.202000061.

[ref15] Ran J.; Jaroniec M.; Qiao S. Z. Cocatalysts in Semiconductor-based Photocatalytic CO2 Reduction: Achievements, Challenges, and Opportunities. Adv. Mater. 2018, 30 (7), 1704649 10.1002/adma.201704649. 29315885

[ref16] Mazzanti S.; Cao S.; ten Brummelhuis K.; Völkel A.; Khamrai J.; Sharapa D. I.; Youk S.; Heil T.; Tarakina N. V.; Strauss V.; Ghosh I.; König B.; Oschatz M.; Antonietti M.; Savateev A. All-organic Z-scheme photoreduction of CO2 with water as the donor of electrons and protons. Appl. Catal., B 2021, 285, 119773 10.1016/j.apcatb.2020.119773.

[ref17] Ong W. J.; Tan L. L.; Ng Y. H.; Yong S. T.; Chai S. P. Graphitic Carbon Nitride (g-C3N4)-Based Photocatalysts for Artificial Photosynthesis and Environmental Remediation: Are We a Step Closer To Achieving Sustainability?. Chem. Rev. 2016, 116 (12), 7159–329. 10.1021/acs.chemrev.6b00075. 27199146

[ref18] Zou Y.; Xiao K.; Qin Q.; Shi J. W.; Heil T.; Markushyna Y.; Jiang L.; Antonietti M.; Savateev A. Enhanced Organic Photocatalysis in Confined Flow through a Carbon Nitride Nanotube Membrane with Conversions in the Millisecond Regime. ACS Nano 2021, 15 (4), 6551–6561. 10.1021/acsnano.0c09661. 33822587 PMC8155341

[ref19] Savateev A.; Ghosh I.; Konig B.; Antonietti M. Photoredox Catalytic Organic Transformations using Heterogeneous Carbon Nitrides. Angew. Chem., Int. Ed. 2018, 57 (49), 15936–15947. 10.1002/anie.201802472. 30066478

[ref20] Khamrai J.; Ghosh I.; Savateev A.; Antonietti M.; König B. Photo-Ni-Dual-Catalytic C(sp2)–C(sp3) Cross-Coupling Reactions with Mesoporous Graphitic Carbon Nitride as a Heterogeneous Organic Semiconductor Photocatalyst. ACS Catal. 2020, 10 (6), 3526–3532. 10.1021/acscatal.9b05598.

[ref21] Majek M.; Neumeier M.; Jacobi von Wangelin A. Aromatic Chlorosulfonylation by Photoredox Catalysis. ChemSusChem 2017, 10 (1), 151–155. 10.1002/cssc.201601293. 27863070

[ref22] Markushyna Y.; Smith C. A.; Savateev A. Organic Photocatalysis: Carbon Nitride Semiconductors vs. Molecular Catalysts. Eur. J. Org. Chem. 2020, 2020 (10), 1294–1309. 10.1002/ejoc.201901112.

[ref23] Savateev A.; Antonietti M. Ionic Carbon Nitrides in Solar Hydrogen Production and Organic Synthesis: Exciting Chemistry and Economic Advantages. ChemCatChem 2019, 11 (24), 6166–6176. 10.1002/cctc.201901076.

[ref24] Markushyna Y.; Völkel A.; Savateev A.; Antonietti M.; Filonenko S. One-pot photocalalytic reductive formylation of nitroarenes via multielectron transfer by carbon nitride in functional eutectic medium. J. Catal. 2019, 380, 186–194. 10.1016/j.jcat.2019.10.010.

[ref25] Kurpil B.; Markushyna Y.; Savateev A. Visible-Light-Driven Reductive (Cyclo)Dimerization of Chalcones over Heterogeneous Carbon Nitride Photocatalyst. ACS Catal. 2019, 9 (2), 1531–1538. 10.1021/acscatal.8b04182.

[ref26] Markushyna Y.; Lamagni P.; Catalano J.; Lock N.; Zhang G.; Antonietti M.; Savateev A. Advantages in Using Inexpensive CO2 To Favor Photocatalytic Oxidation of Benzylamines. ACS Catal. 2020, 10 (13), 7336–7342. 10.1021/acscatal.0c02176.

[ref27] Mazzanti S.; Manfredi G.; Barker A. J.; Antonietti M.; Savateev A.; Giusto P. Carbon Nitride Thin Films as All-In-One Technology for Photocatalysis. ACS Catal. 2021, 11 (17), 11109–11116. 10.1021/acscatal.1c02909.

[ref28] Savateev A.; Tarakina N. V.; Strauss V.; Hussain T.; ten Brummelhuis K.; Sánchez Vadillo J. M.; Markushyna Y.; Mazzanti S.; Tyutyunnik A. P.; Walczak R.; Oschatz M.; Guldi D. M.; Karton A.; Antonietti M. Potassium Poly(Heptazine Imide): Transition Metal-Free Solid-State Triplet Sensitizer in Cascade Energy Transfer and [3 + 2]-cycloadditions. Angew. Chem., Int. Ed. 2020, 59 (35), 15061–15068. 10.1002/anie.202004747. PMC7496904 32412175

[ref29] Markushyna Y.; Teutloff C.; Kurpil B.; Cruz D.; Lauermann I.; Zhao Y.; Antonietti M.; Savateev A. Halogenation of aromatic hydrocarbons by halide anion oxidation with poly(heptazine imide) photocatalyst. Appl. Catal., B 2019, 248, 211–217. 10.1016/j.apcatb.2019.02.016.

[ref30] Chen Z.; Pronkin S.; Fellinger T.-P.; Kailasam K.; Vilé G.; Albani D.; Krumeich F.; Leary R.; Barnard J.; Thomas J. M.; Pérez-Ramírez J.; Antonietti M.; Dontsova D. Merging Single-Atom-Dispersed Silver and Carbon Nitride to a Joint Electronic System via Copolymerization with Silver Tricyanomethanide. ACS Nano 2016, 10 (3), 3166–3175. 10.1021/acsnano.5b04210. 26863408

[ref31] Liu J.; Zou Y.; Cruz D.; Savateev A.; Antonietti M.; Vilé G. Ligand–Metal Charge Transfer Induced via Adjustment of Textural Properties Controls the Performance of Single-Atom Catalysts during Photocatalytic Degradation. ACS Appl. Mater. Interfaces 2021, 13 (22), 25858–25867. 10.1021/acsami.1c02243. 34028257 PMC8289176

[ref32] Colombari F. M.; da Silva M. A. R.; Homsi M. S.; de Souza B. R. L.; Araujo M.; Francisco J. L.; da Silva G. T. S. T.; Silva I. F.; de Moura A. F.; Teixeira I. F. Graphitic carbon nitrides as platforms for single-atom photocatalysis. Faraday Discuss. 2021, 227 (0), 306–320. 10.1039/C9FD00112C. 33305778

[ref33] Sahoo S. K.; Teixeira I. F.; Naik A.; Heske J.; Cruz D.; Antonietti M.; Savateev A.; Kühne T. D. Photocatalytic Water Splitting Reaction Catalyzed by Ion-Exchanged Salts of Potassium Poly(heptazine imide) 2D Materials. J. Phys. Chem. C 2021, 125 (25), 13749–13758. 10.1021/acs.jpcc.1c03947. PMC8256424 34239658

[ref34] da Silva M. A. R.; Silva I. F.; Xue Q.; Lo B. T. W.; Tarakina N. V.; Nunes B. N.; Adler P.; Sahoo S. K.; Bahnemann D. W.; Salas N. L.; Savateev A.; Ribeiro C.; Kühne T. D.; Antonietti M.; Teixeira I. F. Sustainable Oxidation Catalysis Supported by Light: Fe-Poly (heptazine imide) as a Heterogeneous Single-Atom Photocatalyst. Applied Catalysis B: Environmental 2022, 304, 120965 10.1016/j.apcatb.2021.120965.

[ref35] Markushyna Y.; Lamagni P.; Teutloff C.; Catalano J.; Lock N.; Zhang G.; Antonietti M.; Savateev A. Green radicals of potassium poly(heptazine imide) using light and benzylamine. J. Mater. Chem. A 2019, 7 (43), 24771–24775. 10.1039/C9TA09500D.

[ref36] Savateev A.; Markushyna Y.; Schüßlbauer C. M.; Ullrich T.; Guldi D. M.; Antonietti M. Unconventional Photocatalysis in Conductive Polymers: Reversible Modulation of PEDOT:PSS Conductivity by Long-lived Poly(Heptazine Imide) Radicals. Angew. Chem., Int. Ed. 2021, 60 (13), 7436–7443. 10.1002/anie.202014314. PMC8048452 33259655

[ref37] Markushyna Y.; Schüßlbauer C. M.; Ullrich T.; Guldi D. M.; Antonietti M.; Savateev A. Chromoselective Synthesis of Sulfonyl Chlorides and Sulfonamides with Potassium Poly(heptazine imide) Photocatalyst. Angew. Chem., Int. Ed. 2021, 60 (37), 20543–20550. 10.1002/anie.202106183. PMC8457082 34223699

[ref38] Hari D. P.; Schroll P.; Konig B. Metal-free, visible-light-mediated direct C-H arylation of heteroarenes with aryl diazonium salts. J. Am. Chem. Soc. 2012, 134 (6), 2958–61. 10.1021/ja212099r. 22296099

[ref39] Ghosh I.; Khamrai J.; Savateev A.; Shlapakov N.; Antonietti M.; König B. Organic semiconductor photocatalyst can bifunctionalize arenes and heteroarenes. Science 2019, 365 (6451), 360–366. 10.1126/science.aaw3254. 31346061

[ref40] Chen Z.; Savateev A.; Pronkin S.; Papaefthimiou V.; Wolff C.; Willinger M. G.; Willinger E.; Neher D.; Antonietti M.; Dontsova D. The Easier the Better” Preparation of Efficient Photocatalysts—Metastable Poly(heptazine imide) Salts. Adv. Mater. 2017, 29 (32), 1700555 10.1002/adma.201700555. 28632318

[ref41] Andrieux C. P.; Pinson J. The standard redox potential of the phenyl radical/anion couple. J. Am. Chem. Soc. 2003, 125 (48), 14801–6. 10.1021/ja0374574. 14640655

