# Supplementary material for: Synthesis of Sulfonyl Chlorides from Aryldiazonium Salts Mediated by a Heterogeneous Potassium Poly(heptazine imide) Photocatalyst
Source: ACS Org Inorg Au. 2021 Dec 13;2(2):153–8. doi: 10.1021/acsorginorgau.1c00038 (PMC9955386; doi:10.1021/acsorginorgau.1c00038)
Supplement: Supplementary file 1 — gg1c00038_si_001.pdf [file gg1c00038_si_001.pdf]

# Synthesis of sulfonyl chlorides from aryldiazonium salts mediated by heterogeneous potassium poly(heptazine imide) photocatalyst

Yevheniia Markushyna,* Markus Antonietti and Aleksandr Savateev*

Max-Planck Institute of Colloids and Interfaces, Department of Colloid Chemistry, Am Mühlenberg 1, 14476 Potsdam, Germany

# Supporting information

## Chemicals

All chemicals were used without purification. Acetonitrile (≥99.8%, Sigma Aldrich), chloroform-*d* (99.8 atom% D, Sigma Aldrich), dichloromethane (≥99.9%, Sigma Aldrich), dimethylsulfoxide-$d_6$ (99.5 atom % D, Sigma Aldrich), thionyl chloride (99%, Sigma Aldrich), aniline (99%, Alfa Aesar), 4-bromoaniline (99%, TCI), 4-chloroaniline (99%, TCI), p-anisidine (99%, Alfa Aesar), 4-aminobenzonitrile (98%, TCI), methyl 4-aminobenzoate (98%, Sigma Aldrich), 4-nitroaniline (99%, Sigma Aldrich).

## Characterization methods

***$^1$H and $^{13}$C NMR*** spectra were recorded on an Agilent 400 MHz (at 400 MHz for Protons and 101 MHz for Carbon-13). NMR spectra were recorded in $CDCl_3$ or DMSO-d6. The chemical shifts are reported in ppm relative to the residual signal of $CHCl_3$ (7.26 ppm in $^1$H NMR, 77.16 ppm for $^{13}$C NMR) or DMSO (2.5 ppm in $^1$H NMR, 39.52 ppm for $^{13}$C NMR).

***GC-MS.*** Agilent 6890 Network GC System coupled with Agilent 5975 Inert Mass Selective detector (electron ionization) were used for reaction mixture composition analysis and to obtain mass spectra of the products.

***Fourier transform infrared (FT-IR)*** spectra were recorded on Thermo Scientific Nicolet iD5 spectrometer.

***Optical absorbance*** spectra of powders were measured on a Shimadzu UV 2600 equipped with an integrating sphere in diffuse reflectance mode.

***Steady-state fluorescence*** spectra were measured on Jasco FP-8300 fluorescence spectrometer.

***Powder X-Ray diffraction*** patterns were measured on a Bruker D8 Advance diffractometer equipped with a scintillation counter detector with CuKα radiation (λ = 0.15418 nm) applying 2θ step size of 0.05° and counting time of 3 s per step.

***Nitrogen adsorption/desorption*** measurements were performed after degassing the samples at 150 °C for 20 hours using a Quantachrome Quadrasorb SI-MP porosimeter at 77.4 K. The specific surface areas were calculated by applying the Brunauer-Emmett-Teller (BET) model to adsorption isotherms for $0.05 < p/p_0 < 0.3$ using the QuadraWin 5.11 software package.

***Scanning electron microscopy (SEM)*** images were obtained on JSM-7500F (JEOL) microscope. Energy disperse X-ray (EDX) analysis and morphology observation by scanning electron microscope (SEM) were performed using a Link ISIS-300 system (Oxford Microanalysis Group).

***X-ray photoelectron spectroscopy (XPS)*** measurements were carried out in an ultrahigh vacuum (UHV) spectrometer equipped with a VSW Class WA hemispherical electron analyzer. A dual anode Al Kα X-ray source (1486.6 eV) was used as incident radiation. Survey and high resolution spectra were recorded in constant pass energy mode (44 and 22 eV, respectively). During the UPS (He I excitation energy hv=21.23 eV) measurements a bias of 15.32 V was applied to the sample, in order to avoid interference of the spectrometer threshold in the UP spectra. The values of the valence band maximum (VBM) are determined by fitting a straight line into the leading edge.

***TEM measurements*** were acquired using a double-corrected JEOL JEM-ARM200F, equipped with a cold field emission gun and a Gatan GIF Quantum.

## Photocatalysts preparation

### *K-PHI preparation*

K-PHI was prepared according to the literature procedure.[1] A mixture of lithium chloride (3.71 g), potassium chloride (4.54 g) and 5-aminotetrazole (1.65 g) was ground in ball mill for 5 min at the shaking rate 25 $s^{-1}$. Reaction mixtures were transferred into porcelain crucibles and covered with lids. Crucibles were placed in the oven and heated under constant nitrogen flow (15 $L \cdot min^{-1}$) and atmospheric pressure at a following temperature regime: heating from room temperature to 550 °C for 4 hours, annealing at 550 °C for 4 hours. After completion of the heating program, the crucibles were allowed to cool slowly to room temperature under nitrogen flow. The crude products were removed from the crucibles, washed with deionized water (100 mL) for 3 hours in order to remove salts, then filtered, extensively washed with deionized water and dried in a vacuum oven (20 mbar) at 50 °C for 15 h.

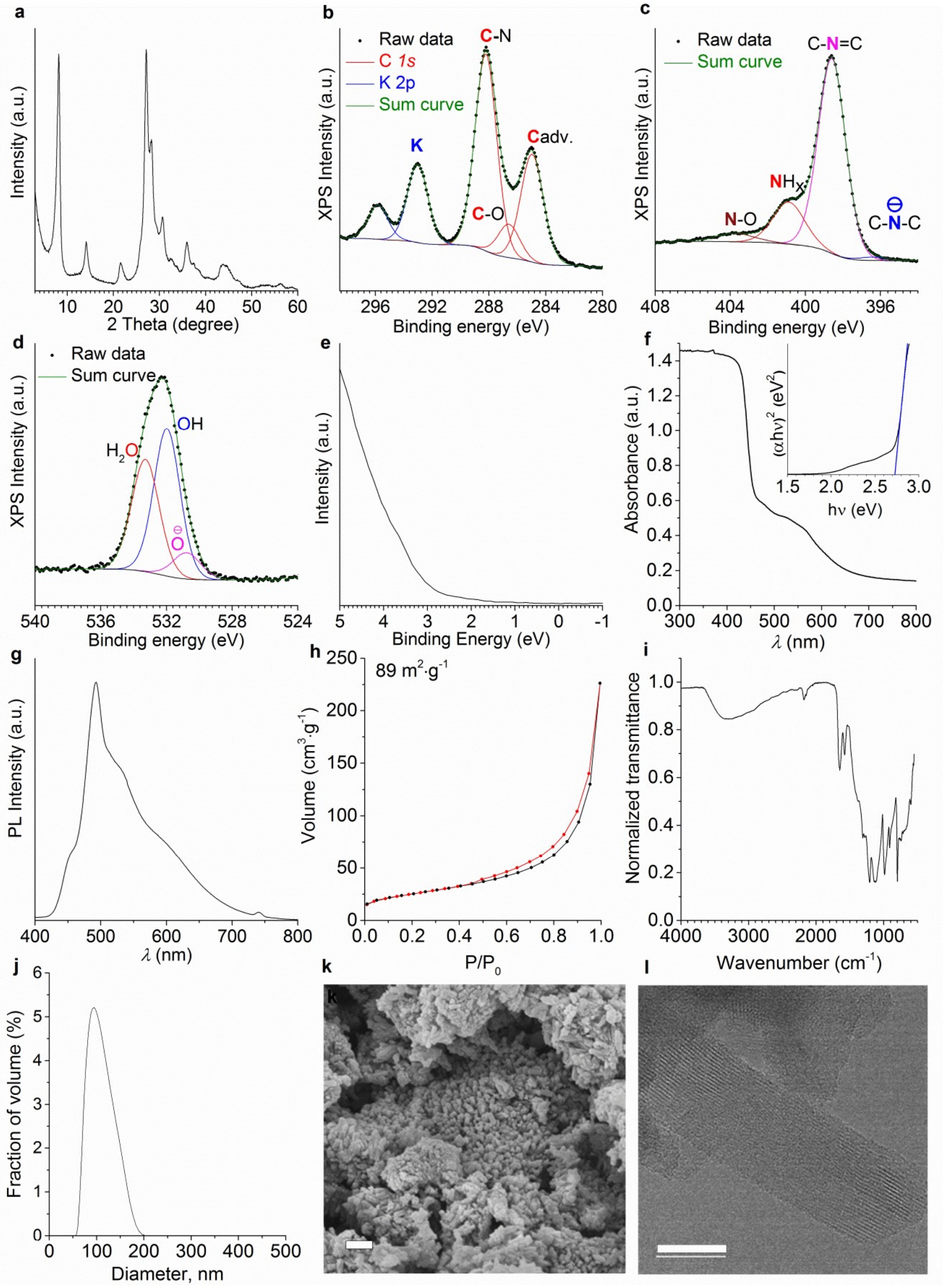

**Figure S1**. K-PHI characterization. a) PXRD pattern of K-PHI; b) XPS C 1s and K 2p spectra of K-PHI; c) XPS N 1s spectrum of K-PHI; d) XPS O 1s spectrum of K-PHI; e) UPS spectrum of K-PHI; f) UV-vis absorption spectrum of K-PHI with Tauc plot as inset assuming that K-PHI is a direct semiconductor; g) PL spectrum of K-PHI obtained upon excitation with 350 nm; h) $N_2$ sorption isotherm measured at 77 K. BET surface area; i) FT-IR spectrum of K-PHI; j) Dynamic light scattering (DLS) analysis of K-PHI suspension in water; k) representative SEM image of K-PHI photocatalyst. Scale bar 200 nm; l) AC-HRTEM image of K-PHI photocatalyst. Scale bar 20 nm. Reproduced from *Nat Commun* 10, 945 (2019).[2]

### *g-CN preparation*

g- CN was prepared according to the following procedure. Dicyandiamide (15 g) was heated to 600 °C with a ramp 2.4 °C under N2 flow. After cooling to room temperature, solid was finely ground in mortar.

### *mpg-CN preparation*

mpg-CN was prepared according to the procedure described in literature.[3] Cyanamide (3.0 g) and Ludox HS-40 (7.5 g) were mixed in a 10 mL glass vial. The mixture was stirred at room temperature for 30 min until cyanamide has completely dissolved. The resultant solution was stirred at +60°C for 16 h until water has completely evaporated. The magnetic stirrer bar was removed and white solid was transferred to the porcelain crucible and heated under $N_2$ flow in the oven. The temperature was increased from room temperature to 550 °C within 4 h and maintained at 550°C for 4 h. The crucible was spontaneously cooled to room temperature. The solid from the crucible was briefly grinded in the mortar and transferred to the polypropylene bottle. A solution of $(NH_4)HF_2$ (0.24 g·mL$^{-1}$, 50 mL) was added and suspension was stirred at room temperature for 24 h. The solid was filtered, thoroughly washed with water, once with ethanol and dried in vacuum (55°C, 20 mbar) overnight.

### *Na-PHI preparation*

Na-PHI was prepared according to the literature procedure. [4] Melamine (1 g) was grinded with NaCl (10 g). Reaction mixture was transferred into a porcelain crucible and covered with lid. Crucible was placed in the oven and heated under constant nitrogen flow (15 L·min$^{-1}$) to 600 °C with a heating rate of 2.3 °C/min, held at 600 °C for 4 hours, then allowed to cool down. The crude product was removed from the crucible, washed with deionized water (100 mL), isolated by filtration, then thoroughly washed with deionized water on the filter (100 mL) and dried in a vacuum oven at 50 °C for 15 h.

## Possible products of the Meerwein type chlorosulfonylation

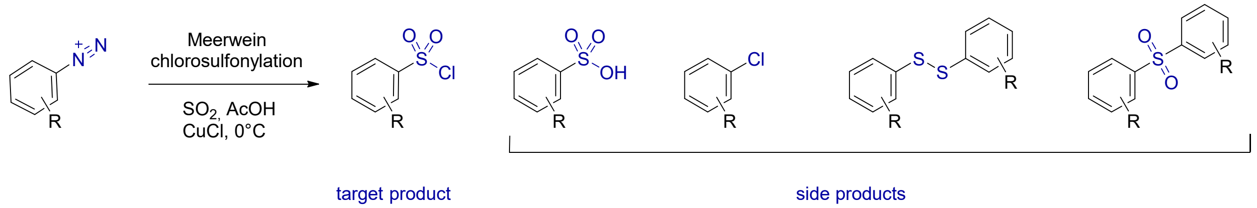


**Scheme S1**. Possible products of the Meerwein type chlorosulfonylation

# Water content in acetonitrile

**Table S1.** Water content in MeCN analyzed by Karl-Fischer titration

| Component | $H_2O$ content, ppm | $V_{component}$, mL | Amount of $H_2O$, µmoL |
|---|---|---|---|
| MeCN | 640 | 1 | 35.5 |
| Anhydrous MeCN | 252 | 1 | 14 |

# Reaction with DABSO

A glass vial was charged with 4-chlorophenyldiazonim tetrafluoroborate **1f** (0.025 mmol), K-PHI (4 mg), acetonitrile (1 mL) and the solution was purged with Ar for 5 min. Then, DABSO (0.125 mmol) and 2M HCl in $Et_2O$ (62 µL, 0.125 mmol) were added. The reaction mixture was stirred under blue LED irradiation (465 nm, 46 mW·cm$^{-2}$) at room temperature for 24 h. After the irradiation solution was concentrated under reduced pressure and analyzed by GC-MS.

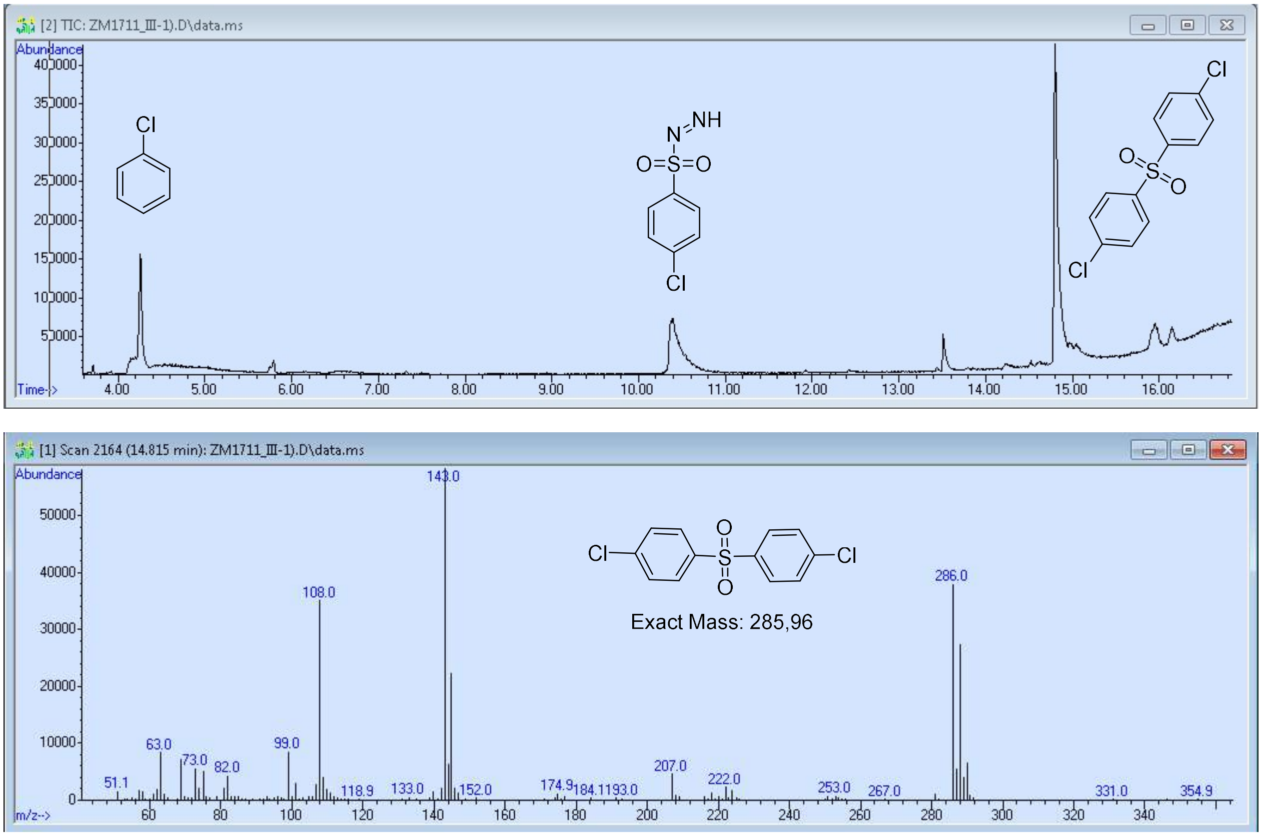


**Figure S2**. GC-MS chromatogram of the reaction mixture prepared with DABSO and MS spectrum of the main product. A peak with retention time of ca. 10.5 min was tentatively assigned to ((4-chlorophenyl)sulfonyl)diazene.[5]

## Synthesis methods

### *Synthesis of arenediazonium tetrafluoroborates* **1**

The diazonium salts have been prepared according to the reported procedure.[6] Typically, a solution of $NaNO_2$ (0.69 g, 10 mmol) was added dropwise to a cooled on the ice bath solution of arylamine (10 mmol) in $HBF_4$ (3.54 mL, 27 mmol) upon stirring. The reaction mixture was stirred on the ice bath for additional 2 h. Precipitate was separated by filtration, dissolved in acetone (150 mL) and maintained at –20°C overnight. Transparent crystals precipitated. Diethylether (200 mL) was added and suspension was maintained at –20°C for 1 h, solid was separated by filtration, washed with cold diethylether (2 times 5 mL) and dried on filter.

*4-bromobenzenediazonium tetrafluoroborate* **1a**

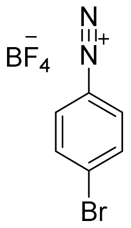


Yield: 86%. $^1$H NMR (400 MHz, DMSO) δ 8.57 (d, *J* = 9.0 Hz, 2H), 8.26 (d, *J* = 9.0 Hz, 2H). $^{13}$C NMR (101 MHz, DMSO) δ 136.59, 134.57, 134.03, 115.76.

*4-methoxybenzenediazonium tetrafluoroborate* **1b**

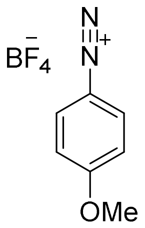


Yield 78%. $^1$H NMR (400 MHz, DMSO) δ 8.61 (d, *J* = 9.4 Hz, 2H), 7.48 (d, *J* = 9.4 Hz, 2H), 4.04 (s, 3H). $^{13}$C NMR (101 MHz, DMSO) δ 168.78, 136.17, 117.30, 103.43, 57.51.

*4-cyanobenzenediazonium tetrafluoroborate* **1c**

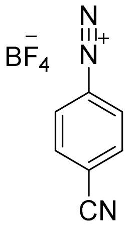


Yield: 88%. $^1$H NMR (400 MHz, DMSO) δ 8.84 (d, *J* = 8.9 Hz, 2H), 8.46 (d, *J* = 8.9 Hz, 2H). $^{13}$C NMR (101 MHz, DMSO) δ 134.87, 133.08, 121.78, 121.13, 116.45.

*4-(methoxycarbonyl)benzenediazonium tetrafluoroborate* **1d**

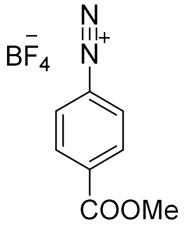


Yield: 65%. $^1$H NMR (400 MHz, DMSO) δ 8.79 (d, *J* = 8.8 Hz, 2H), 8.44 (d, *J* = 8.8 Hz, 2H), 3.95 (s, 3H). $^{13}$C NMR (101 MHz, DMSO) δ 163.89, 137.44, 133.21, 130.69, 120.36, 54.34.

*Benzenediazonium tetrafluoroborate* **1e**

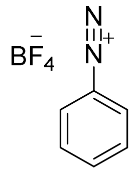


Yield 71%. $^{1}$H NMR (400 MHz, DMSO) δ 8.66 (d, *J* = 7.6 Hz, 2H), 8.26 (t, *J* = 7.6 Hz, 1H), 7.98 (d, 2H). $^{13}$C NMR (101 MHz, DMSO) δ 140.87, 132.71, 131.28, 116.11.

*4-chlorobenzenediazonium tetrafluoroborate* **1f**

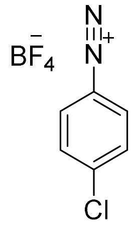


Yield: 85%. $^{1}$H NMR (400 MHz, DMSO) δ 8.69 (d, *J* = 9.0 Hz, 2H), 8.11 (d, *J* = 9.0 Hz, 2H). $^{13}$C NMR (101 MHz, DMSO) δ 146.51, 134.44, 131.64, 114.82.

*4-nitrobenzenediazonium tetrafluoroborate* **1g**

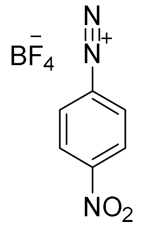


Yield: 97%. $^{1}$H NMR (400 MHz, DMSO) δ 8.92 (d, *J* = 9.3 Hz, 2H), 8.72 (d, *J* = 9.3 Hz, 2H). $^{13}$C NMR (101 MHz, DMSO) δ 153.21, 134.52, 126.07, 121.95.

*4-chloro-3-(trifluoromethyl)benzenediazonium tetrafluoroborate* **1h**

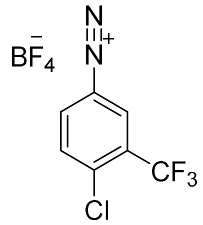


Yield: 72%. $^{1}$H NMR (400 MHz, DMSO) δ 9.33 (d, *J* = 2.4 Hz, 1H), 8.96 (dd, *J* = 8.9, 2.4 Hz, 1H), 8.40 (d, *J* = 8.9 Hz, 1H). $^{13}$C NMR (101 MHz, DMSO) δ 144.16, 137.69, 134.89, 132.87 (q, *J* = 5.8 Hz), 128.77 (q, *J* = 33.6 Hz), 122.40, 116.33.

*3-nitrobenzenediazonium tetrafluoroborate* **1i**

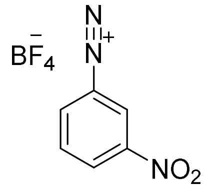


Yield: 91%. $^{1}$H NMR (400 MHz, DMSO) δ 9.61 (s, 1H), 9.06 – 8.92 (m, 2H), 8.24 (t, *J* = 8.4 Hz, 1H). $^{13}$C NMR (101 MHz, DMSO) δ 147.69, 137.95, 132.81, 128.22, 118.28.

*2-fluorobenzenediazonium tetrafluoroborate* **1j**

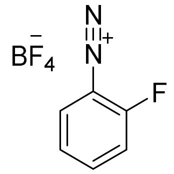


Yield: 78%. $^{1}$H NMR (400 MHz, DMSO) δ 8.74 (m, 1H), 8.38 (m, 1H), 8.01 (t, *J* = 9.2 Hz, 1H), 7.82 (t, *J* = 8.0 Hz, 1H). $^{13}$C NMR (101 MHz, DMSO) δ 160.30 (d, *J* = 273.2 Hz), 144.71 (d, *J* = 9.6 Hz), 133.45 (d, *J* = 4.2 Hz), 127.48 (d, *J* = 3.0 Hz), 118.70 (d, *J* = 15.6 Hz), 105.62 (d, *J* = 12.8 Hz). $^{19}$F NMR (376 MHz, DMSO) δ -102.59, -148.27.

***Synthesis of sulfonylchlorides* 2**

A glass vial was charged with arenediazonium salt **1** (0.025 mmol), and K-PHI (4 mg), acetonitrile (1 mL) (or dichloromethane for phenyldiazonium tetrafluoroborate **1e**) and the solution was purged with Ar for 5 min. Then, water (5 µL, 0.28 mmol) and $SOCl_2$ (19 µL, 0.16 mmol) were added. The reaction mixture was stirred under blue LED irradiation (465 nm, 46 mW·cm$^{-2}$) at room temperature for 24 h. After the irradiation solution was concentrated under reduced pressure and analyzed by NMR.

*4-bromobenzenesulfonyl chloride* **2a**

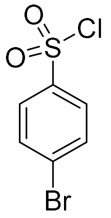


$^1$H NMR (400 MHz, $CDCl_3$) δ 7.91 (d, *J* = 8.8 Hz, 2H), 7.77 (d, *J* = 8.8 Hz, 2H). Spectrum matches reported earlier in reference [7].

*4-methoxybenzenesulfonyl chloride* **2b**

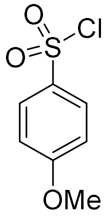


$^1$H NMR (400 MHz, DMSO) δ 7.52 (d, *J* = 8.7 Hz, 2H), 6.86 (d, *J* = 8.7 Hz, 2H), 3.74 (s, 3H). Spectrum matches reported earlier in reference [8].

*4-cyanobenzenesulfonyl chloride* **2c**

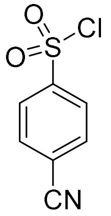


$^1$H NMR (400 MHz, DMSO) δ 7.81 (d, *J* = 8.4 Hz, 2H), 7.74 (d, *J* = 8.4 Hz, 2H).

*4-(methoxycarbonyl)benzene sulfonyl chloride* **2d**

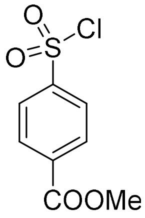


$^1$H NMR (400 MHz, DMSO) δ 7.92 (d, *J* = 8.3 Hz, 2H), 7.71 (d, *J* = 8.3 Hz, 2H), 3.84 (s, 3H).

*4-chlorobenzenesulfonyl chloride* **2f**

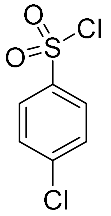

$^1$H NMR (400 MHz, DMSO) δ 7.63 – 7.55 (m, 2H), 7.37 (d, *J* = 8.5 Hz, 2H). Spectrum matches reported earlier in reference [9]

*4-nitrobenzenesulfonyl chloride* **2g**

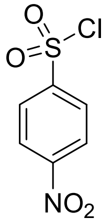

$^1$H NMR (400 MHz, DMSO) δ 8.21 (d, *J* = 8.8 Hz, 2H), 7.83 (d, *J* = 8.8 Hz, 2H).

*4-chloro-3-(trifluoromethyl)benzenesulfonyl chloride* **2h**

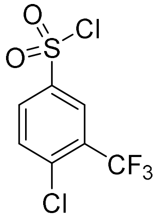

$^1$H NMR (400 MHz, DMSO) δ 7.95 (s, 1H), 7.87 (d, *J* = 10.1 Hz, 1H), 7.71 (d, *J* = 8.3 Hz, 1H).

*3-nitrobenzenesulfonyl chloride* **2i**

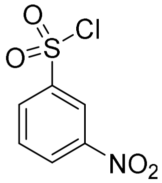

$^1$H NMR (400 MHz, DMSO) δ 8.34 (s, 1H), 8.19 (d, *J* = 9.3 Hz, 1H), 8.02 (d, *J* = 7.6 Hz, 1H), 7.66 (t, *J* = 7.9 Hz, 1H).

*2-fluorobenzenesulfonyl chloride* 2**j**

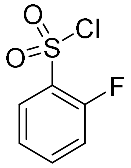

$^1$H NMR (400 MHz, DMSO) δ 7.67 (t, *J* = 7.6 Hz, 1H), 7.43 – 7.31 (m, 1H), 7.16 – 7.07 (m, 2H).

***Supplementary Note 1***

For the case with phenyldiazonium tetrafluoroborate **1e** reaction was carried out in dichloromethane, as it reacts with acetonitrile to form phenylacetamide, $^1H$ NMR spectra of which is presented below.

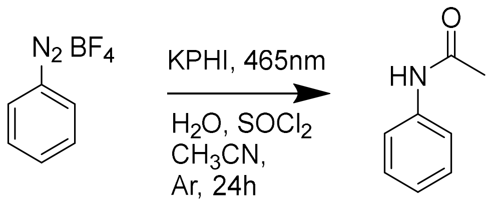


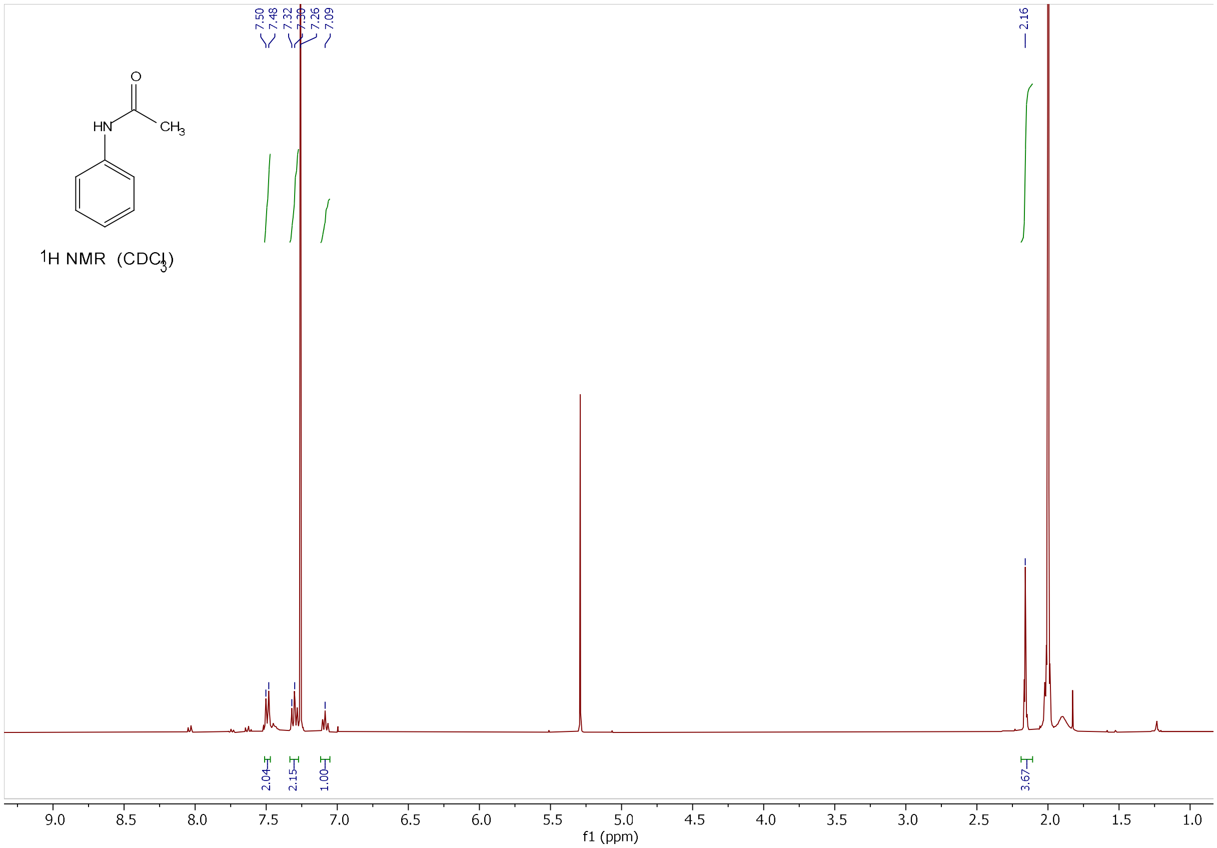


**Figure S3**. Scheme and $^1H$ NMR spectra of the reaction of phenyldiazonium tetrafluoroborate **1e** with acetonitrile with formation of phenylacetamide.

***DMPO-bromophenyl adduct***

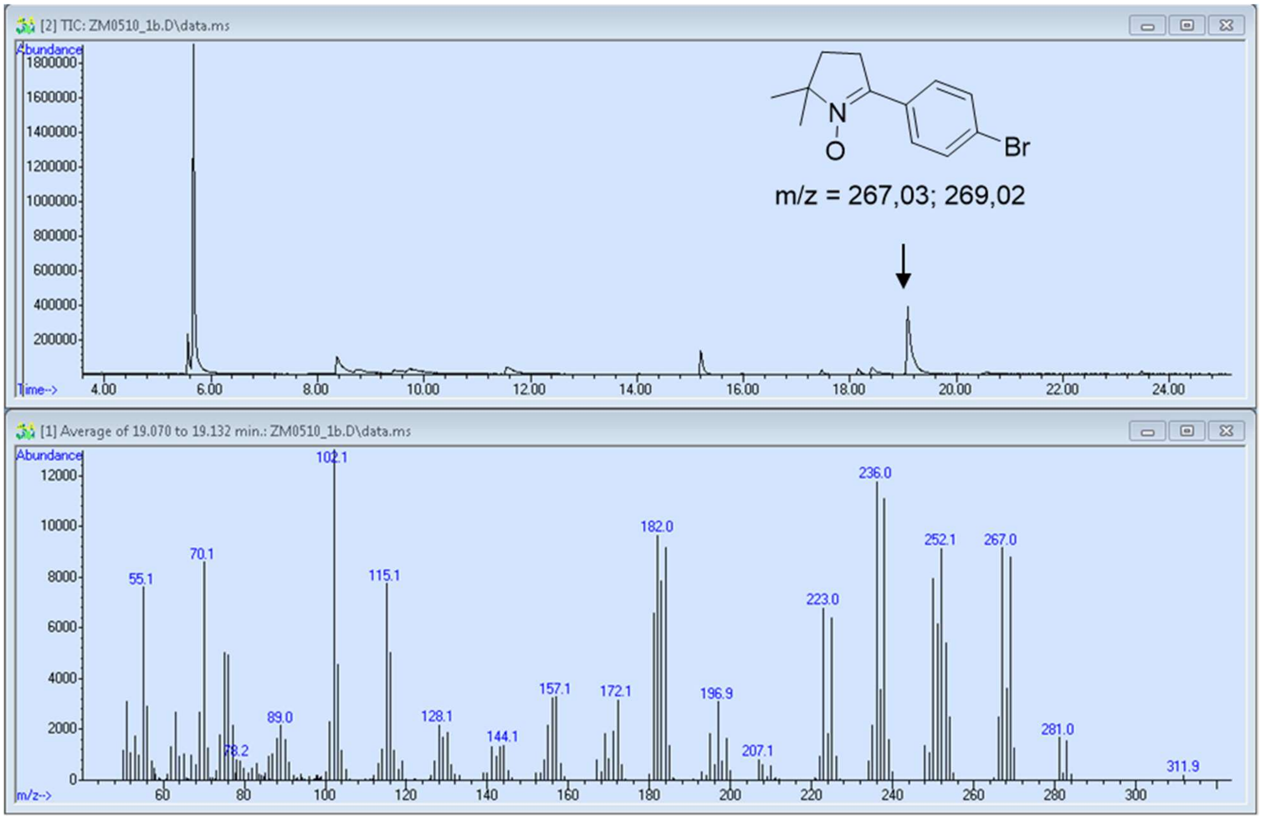


**Figure S4**. GC chromatogram and MS spectra of the DMPO-bromophenyl adduct. Reaction conditions: substrate (0.025 mmol); K-PHI (4 mg); DMPO (0.026 mmol); Ar atmosphere; irradiation with 465 nm LED; 18 h.

# NMR spectra

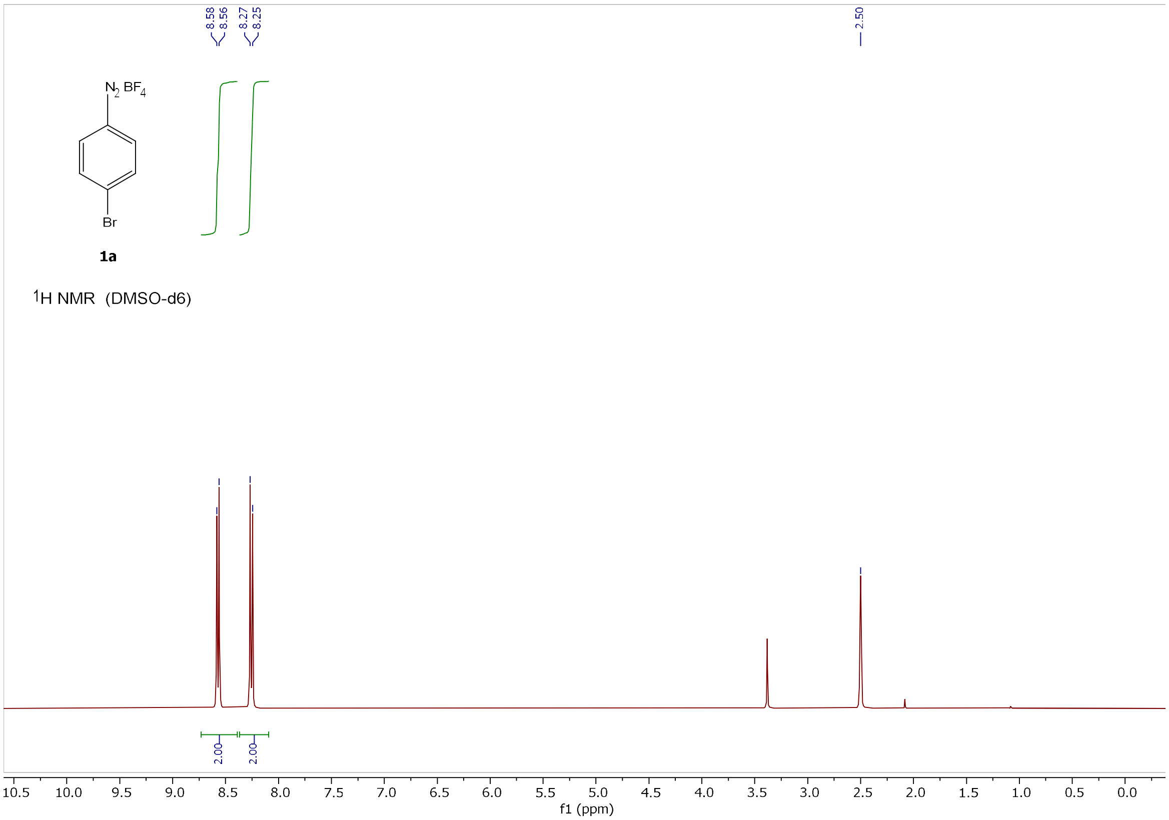
8.58
8.56
8.27
8.25
2.50
$N_2$ $BF_4$
Br
1a
$^1$H NMR (DMSO-d6)
2.00
2.00
f1 (ppm)

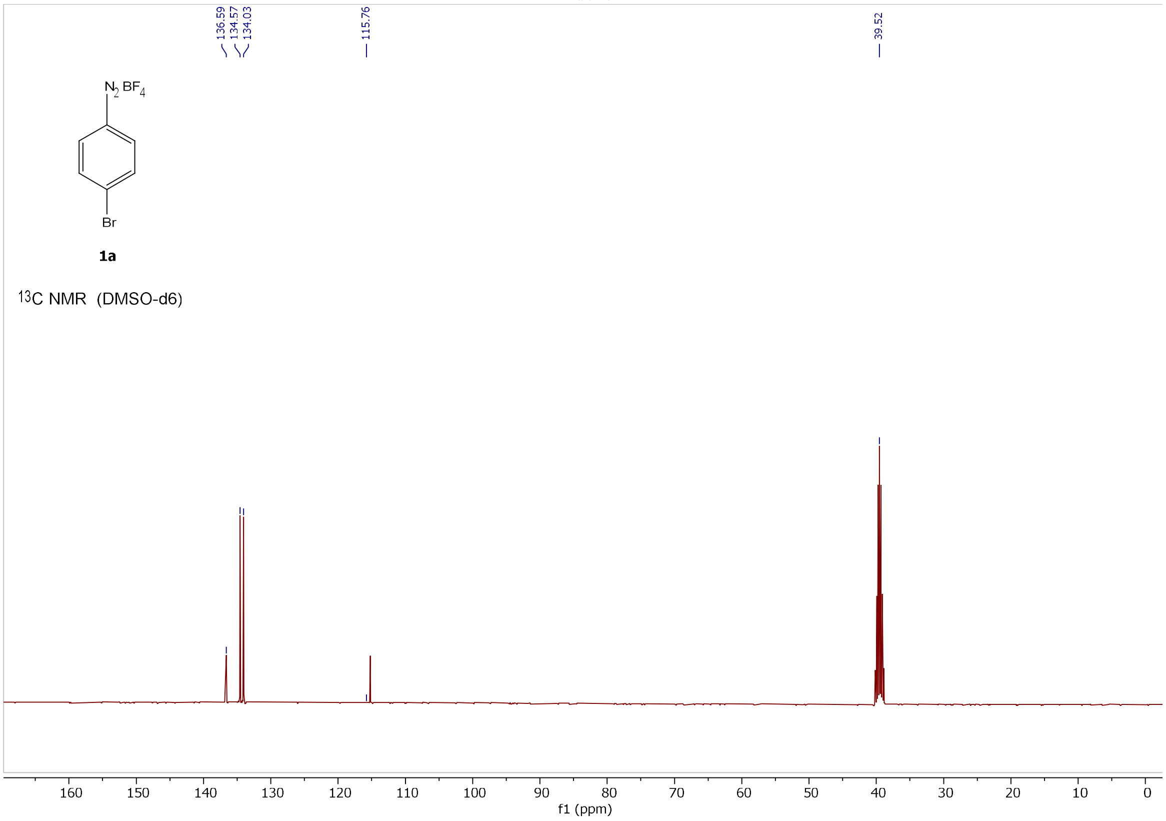
136.59
134.57
134.03
115.76
39.52
$N_2$ $BF_4$
Br
1a
$^{13}$C NMR (DMSO-d6)
f1 (ppm)

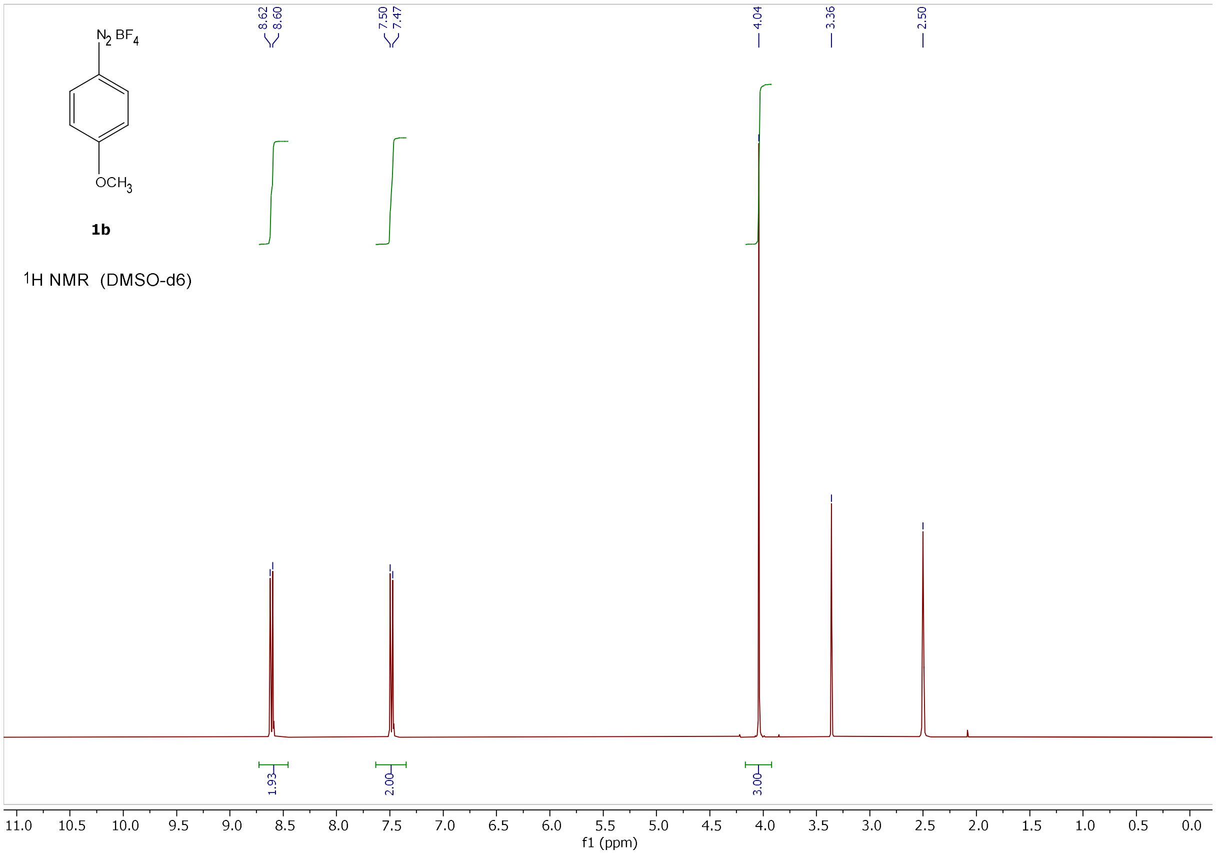

$N_2BF_4$
$OCH_3$
1b
1H NMR (DMSO-d6)
8.62
8.60
7.50
7.47
4.04
3.36
2.50
1.93
2.00
3.00
11.0
10.5
10.0
9.5
9.0
8.5
8.0
7.5
7.0
6.5
6.0
5.5
5.0
4.5
4.0
3.5
3.0
2.5
2.0
1.5
1.0
0.5
0.0
f1 (ppm)


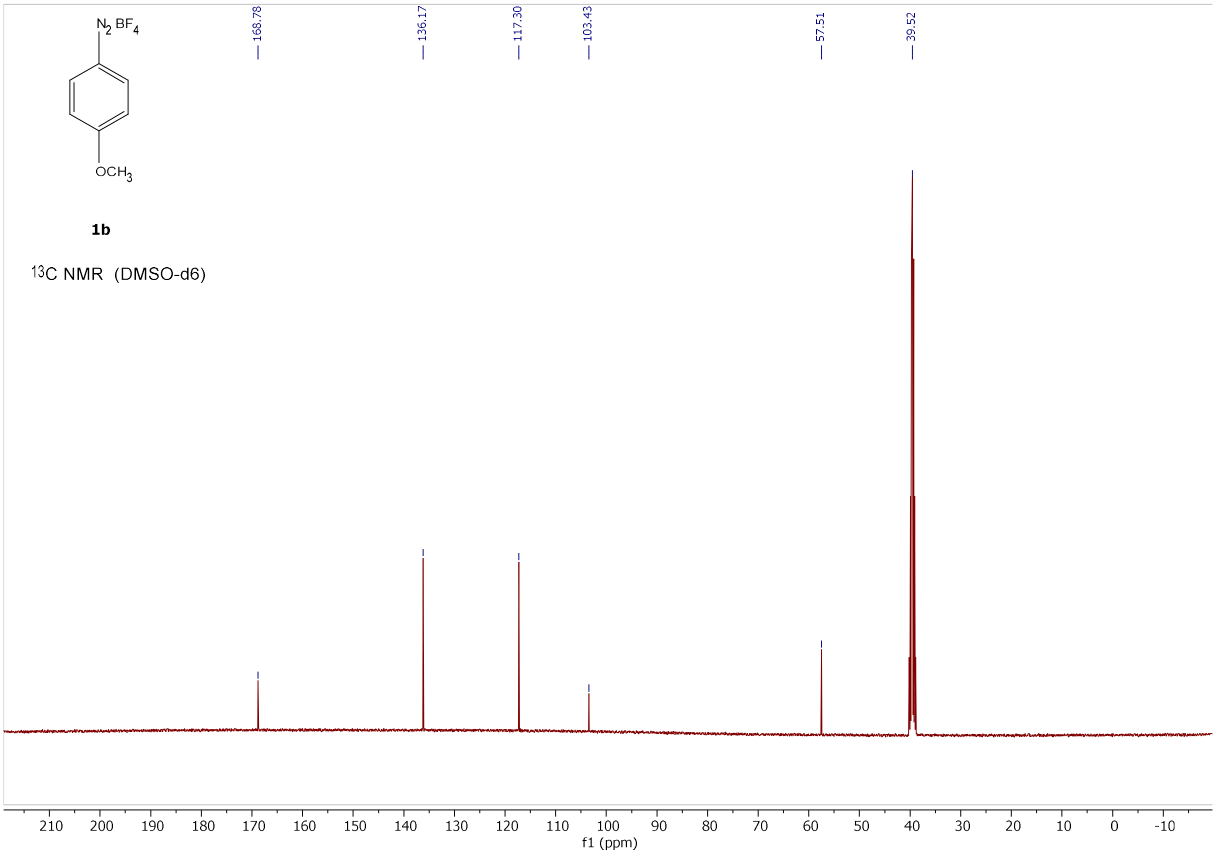

$N_2BF_4$
$OCH_3$
1b
13C NMR (DMSO-d6)
168.78
136.17
117.30
103.43
57.51
39.52
210
200
190
180
170
160
150
140
130
120
110
100
90
80
70
60
50
40
30
20
10
0
-10
f1 (ppm)

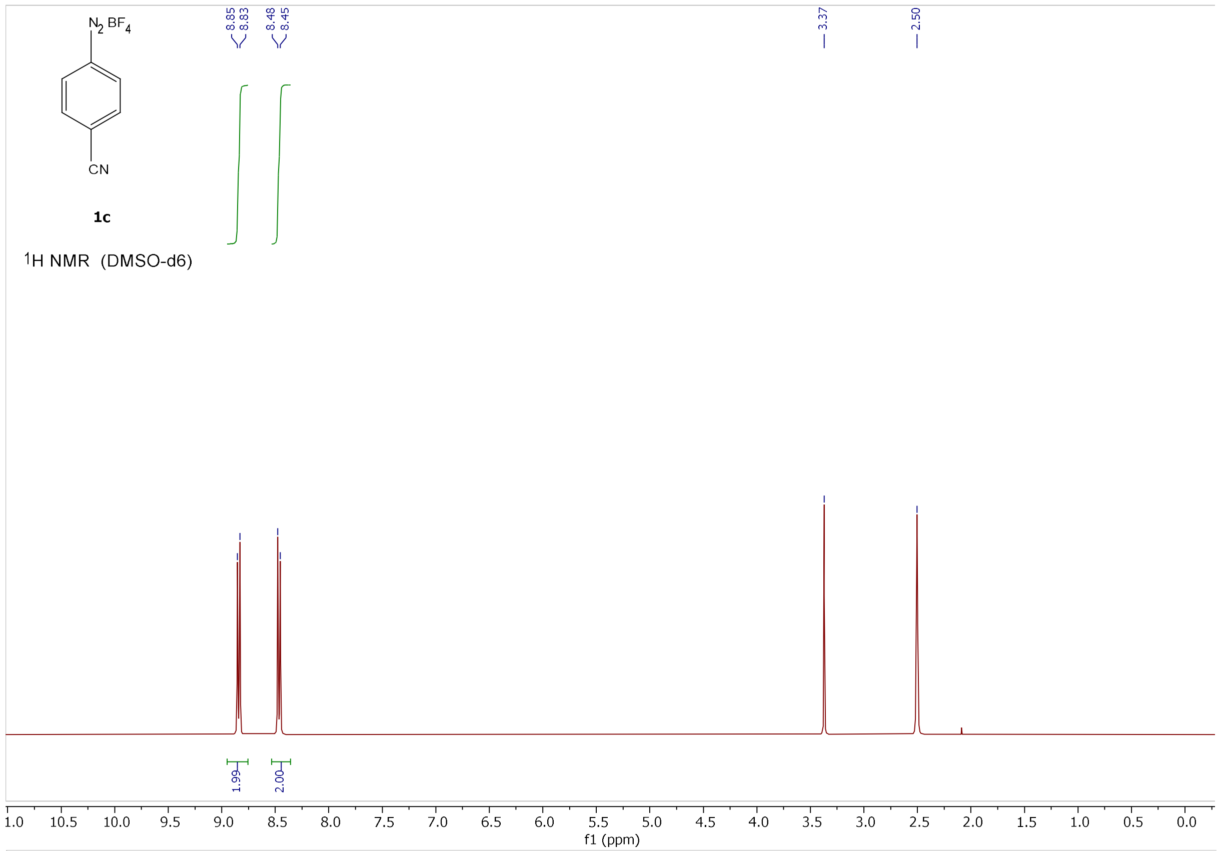

N2 BF4
CN
1c
1H NMR (DMSO-d6)
8.85
8.83
8.48
8.45
3.37
2.50
1.99
2.00
f1 (ppm)


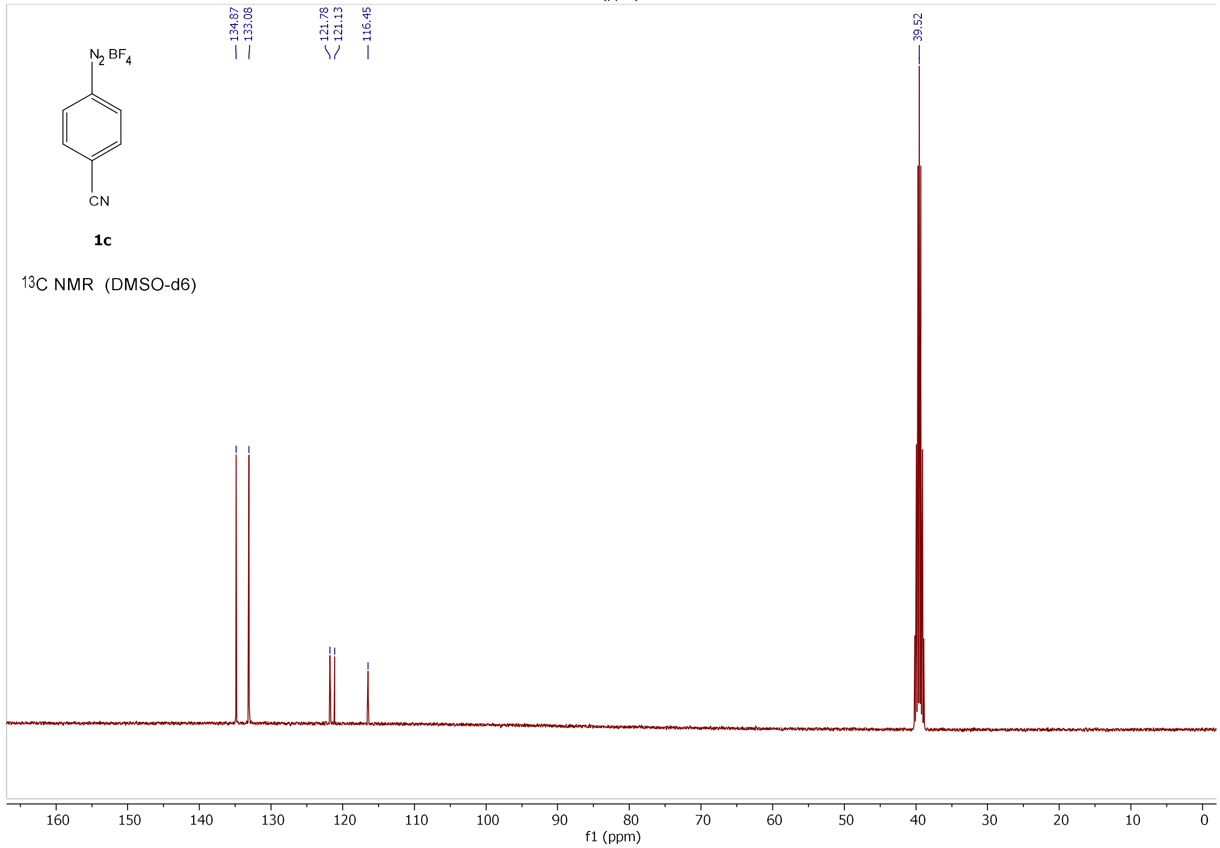

134.87
133.08
121.78
121.13
116.45
39.52
N2 BF4
CN
1c
13C NMR (DMSO-d6)
f1 (ppm)

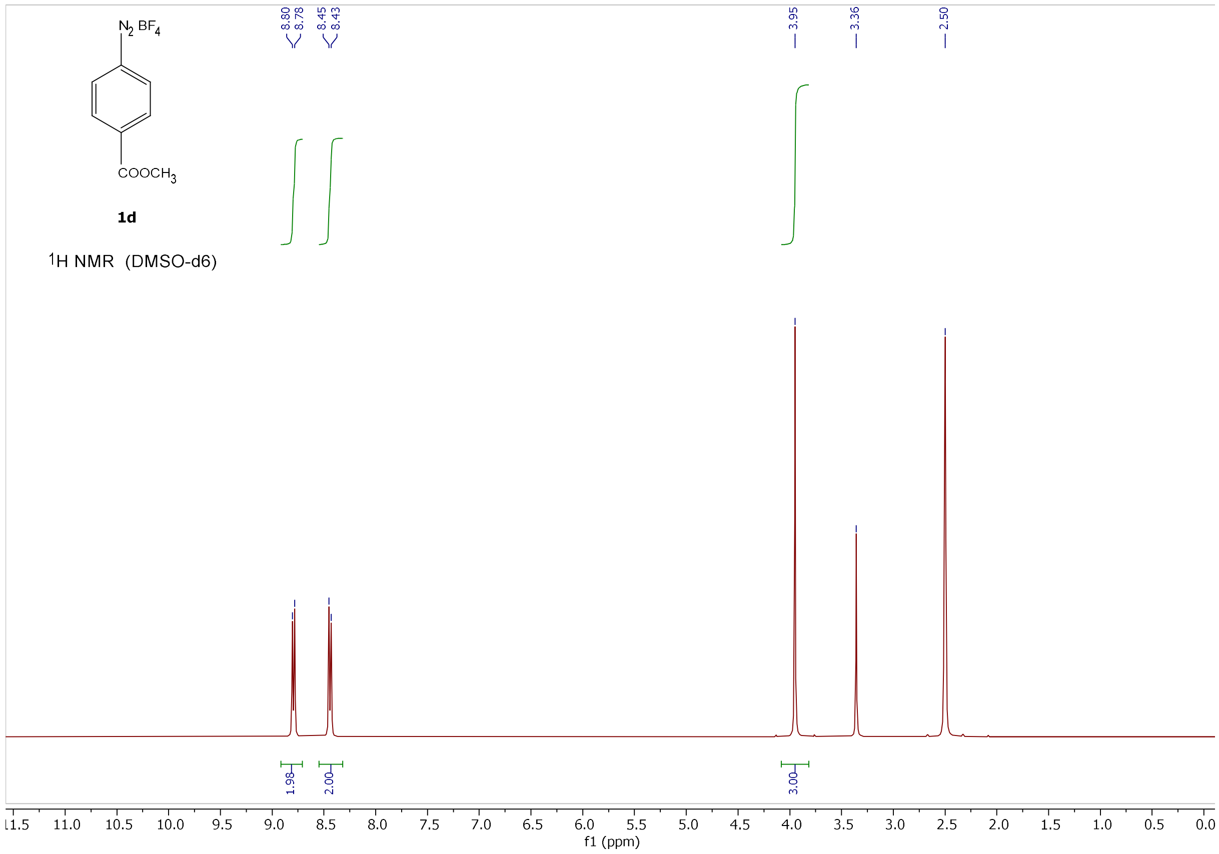

$N_2$ $BF_4$
$COOCH_3$
1d
1H NMR (DMSO-d6)
8.80
8.78
8.45
8.43
3.95
3.36
2.50
1.98
2.00
3.00
f1 (ppm)


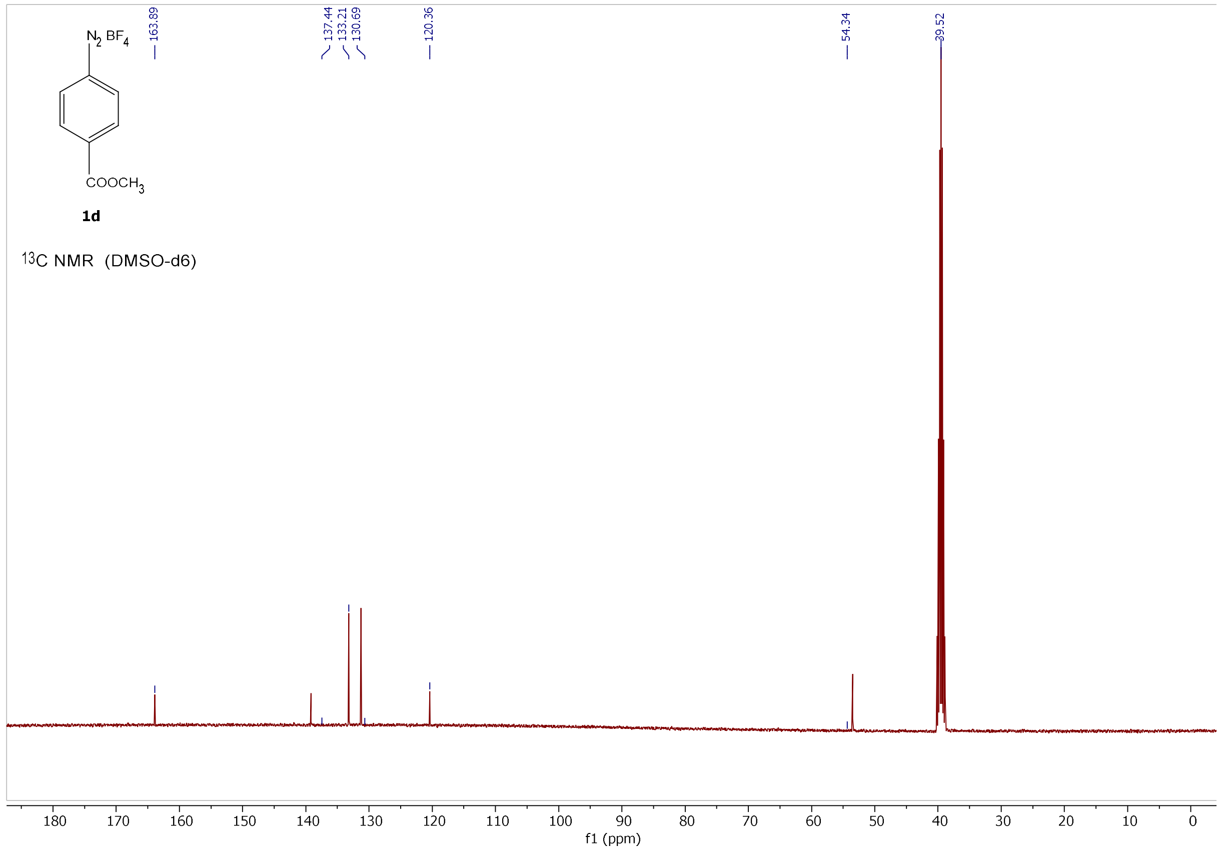

$N_2$ $BF_4$
$COOCH_3$
1d
13C NMR (DMSO-d6)
163.89
137.44
133.21
130.69
120.36
54.34
39.52
f1 (ppm)

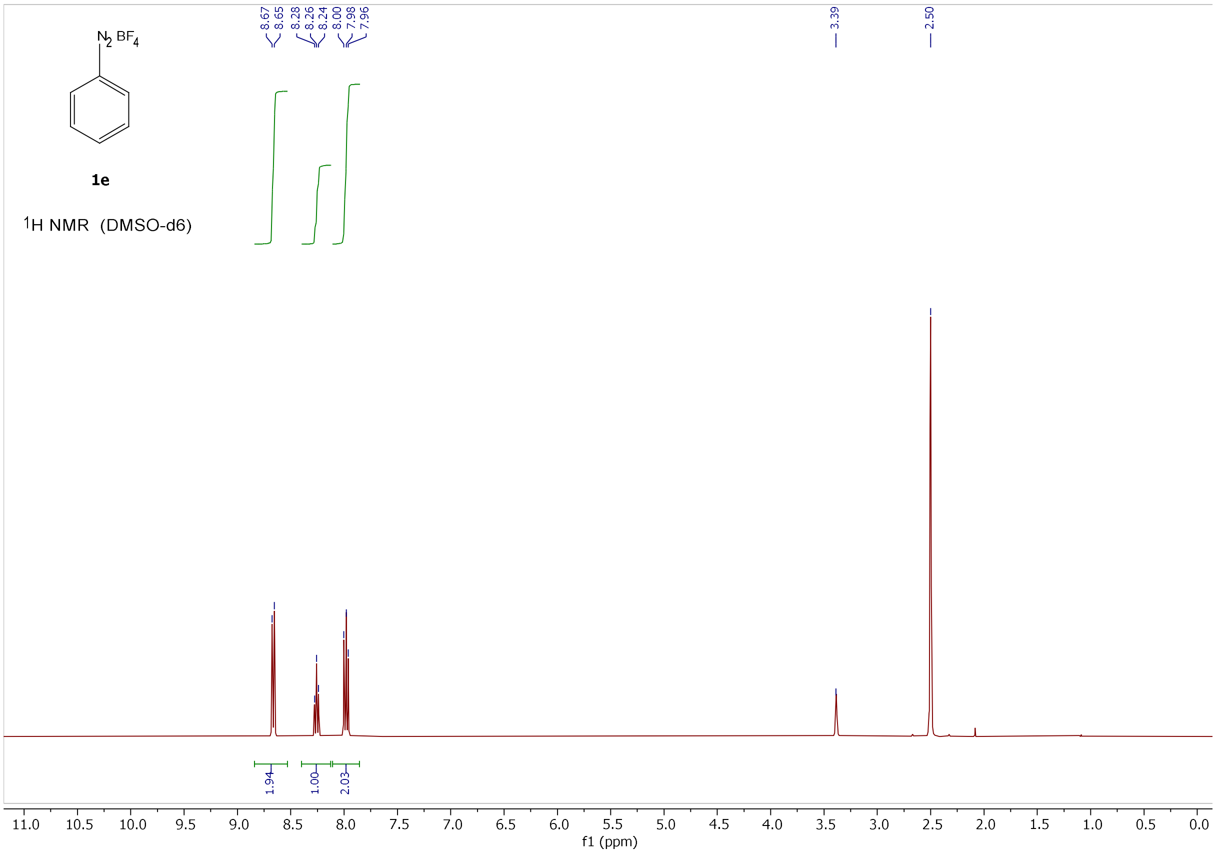

N2 BF4
1e
1H NMR (DMSO-d6)
8.67
8.65
8.28
8.26
8.24
8.00
7.98
7.96
3.39
2.50
1.94
1.00
2.03
11.0 10.5 10.0 9.5 9.0 8.5 8.0 7.5 7.0 6.5 6.0 5.5 5.0 4.5 4.0 3.5 3.0 2.5 2.0 1.5 1.0 0.5 0.0
f1 (ppm)


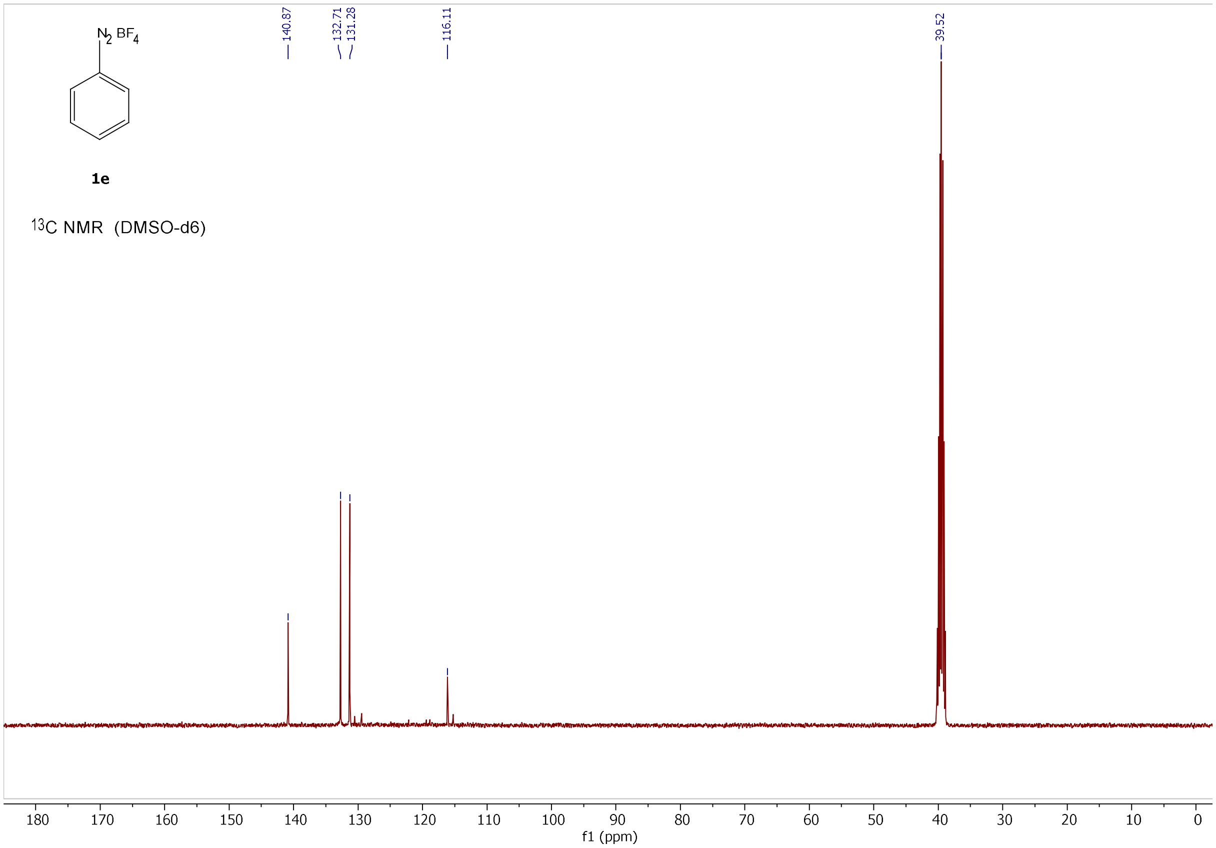

N2 BF4
1e
13C NMR (DMSO-d6)
140.87
132.71
131.28
116.11
39.52
180 170 160 150 140 130 120 110 100 90 80 70 60 50 40 30 20 10 0
f1 (ppm)

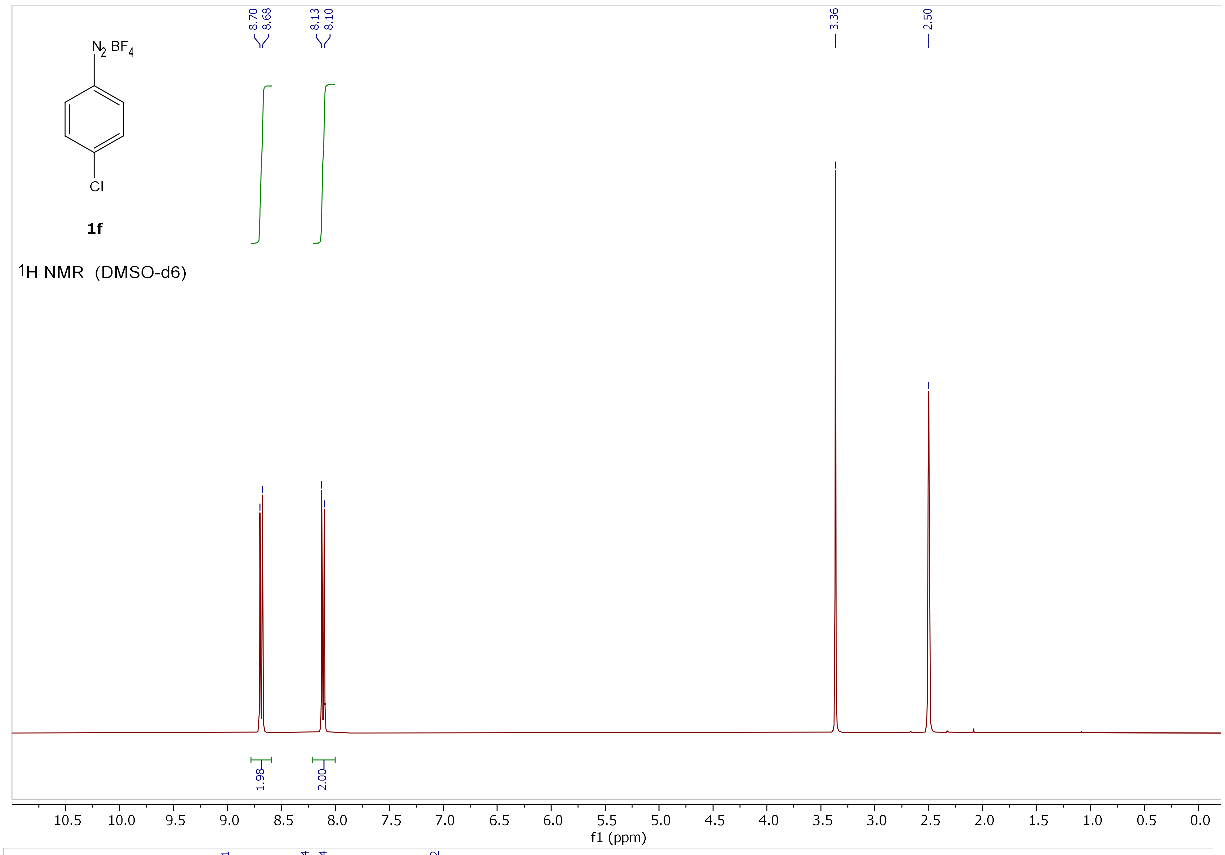
8.70
8.68
8.13
8.10
3.36
2.50
N2 BF4
Cl
1f
1H NMR (DMSO-d6)
1.98
2.00
10.5 10.0 9.5 9.0 8.5 8.0 7.5 7.0 6.5 6.0 5.5 5.0 4.5 4.0 3.5 3.0 2.5 2.0 1.5 1.0 0.5 0.0
f1 (ppm)

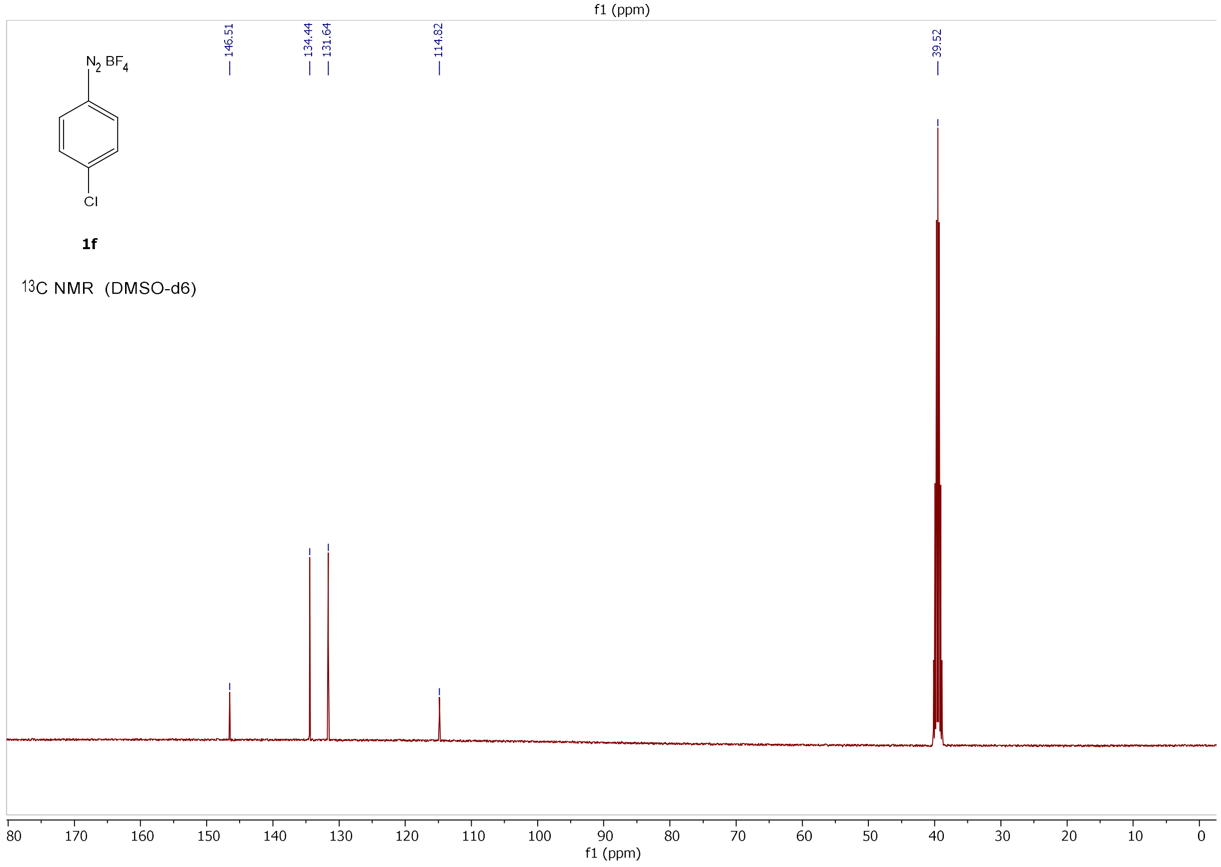
146.51
134.44
131.64
114.82
39.52
N2 BF4
Cl
1f
13C NMR (DMSO-d6)
80 170 160 150 140 130 120 110 100 90 80 70 60 50 40 30 20 10 0
f1 (ppm)

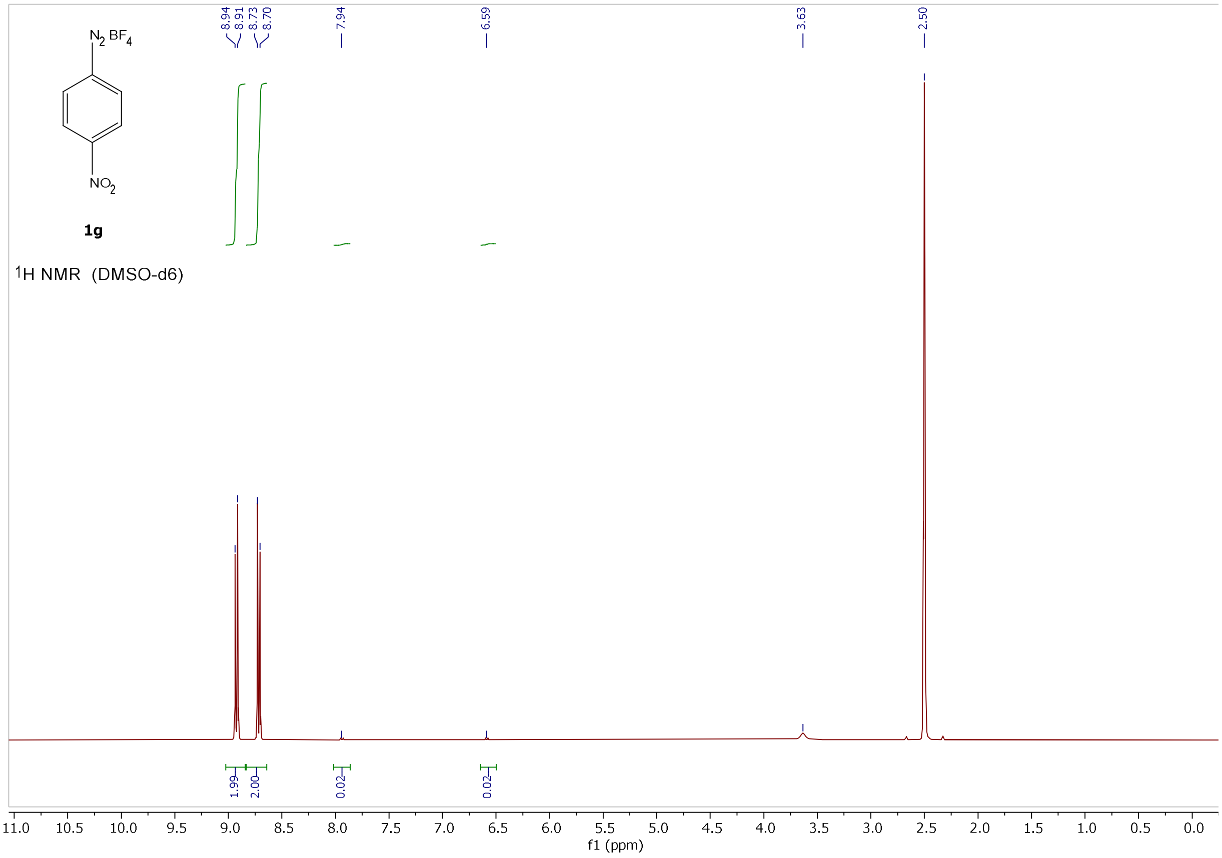

N2 BF4
NO2
1g
1H NMR (DMSO-d6)
8.94
8.91
8.73
8.70
7.94
6.59
3.63
2.50
1.99
2.00
0.02
0.02
11.0 10.5 10.0 9.5 9.0 8.5 8.0 7.5 7.0 6.5 6.0 5.5 5.0 4.5 4.0 3.5 3.0 2.5 2.0 1.5 1.0 0.5 0.0
f1 (ppm)


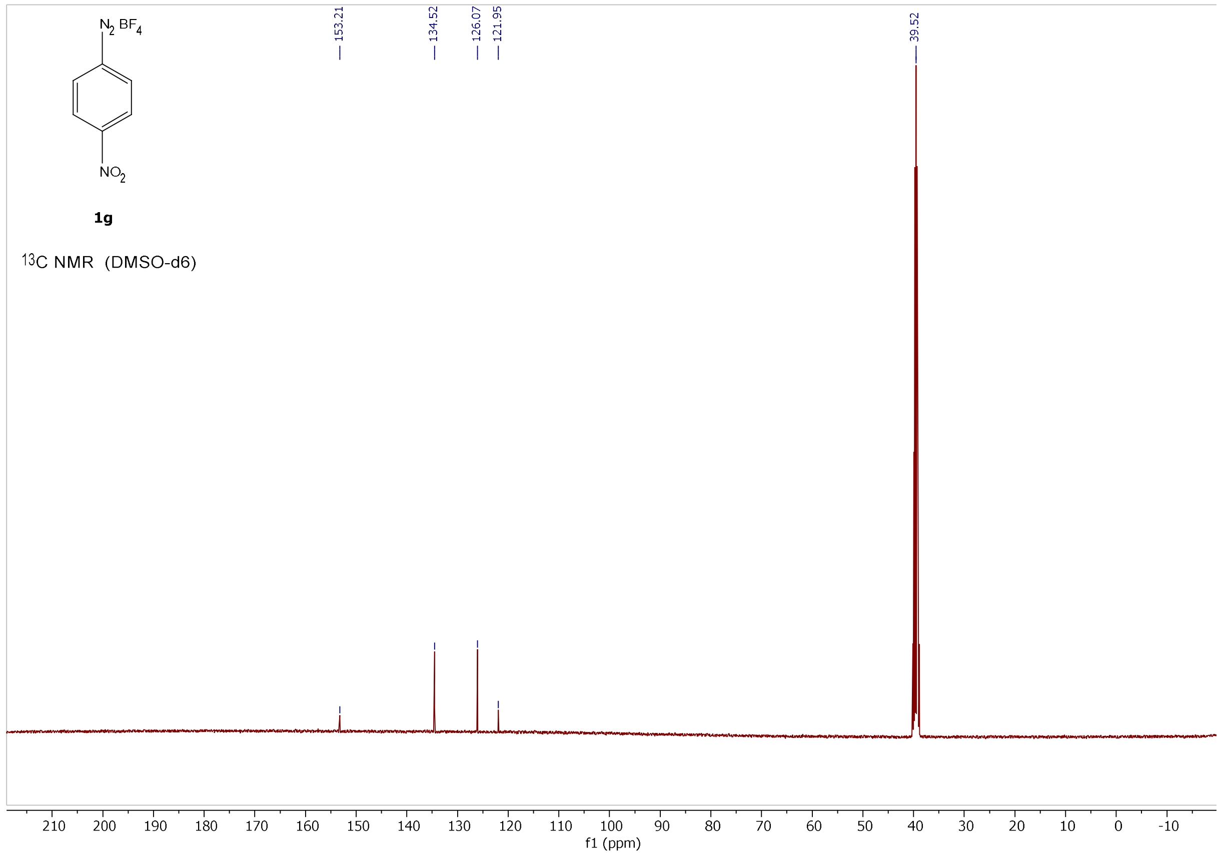

N2 BF4
NO2
1g
13C NMR (DMSO-d6)
153.21
134.52
126.07
121.95
39.52
210 200 190 180 170 160 150 140 130 120 110 100 90 80 70 60 50 40 30 20 10 0 -10
f1 (ppm)

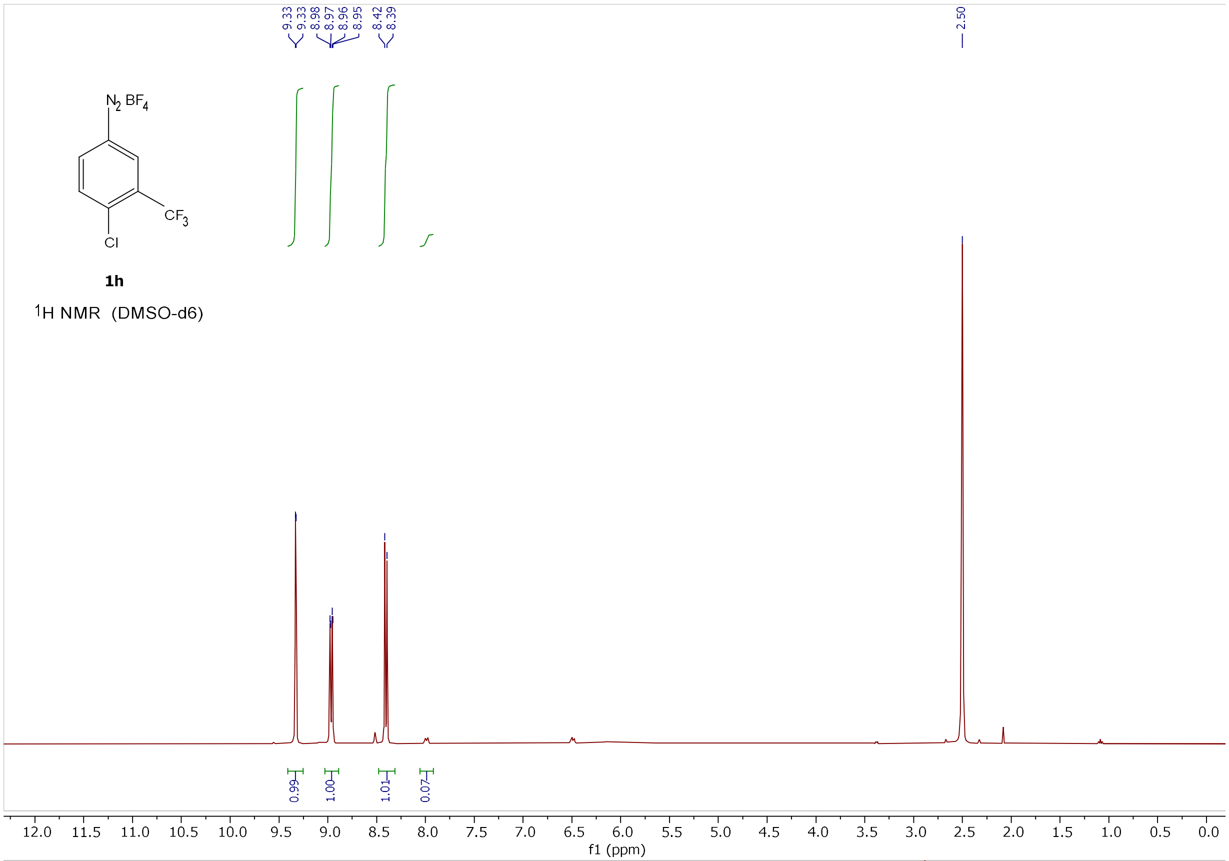
9.33
9.33
8.98
8.97
8.96
8.95
8.42
8.39
2.50
$N_2$ $BF_4$
$CF_3$
Cl
1h
1H NMR (DMSO-d6)
0.99
1.00
1.01
0.07
12.0 11.5 11.0 10.5 10.0 9.5 9.0 8.5 8.0 7.5 7.0 6.5 6.0 5.5 5.0 4.5 4.0 3.5 3.0 2.5 2.0 1.5 1.0 0.5 0.0
f1 (ppm)

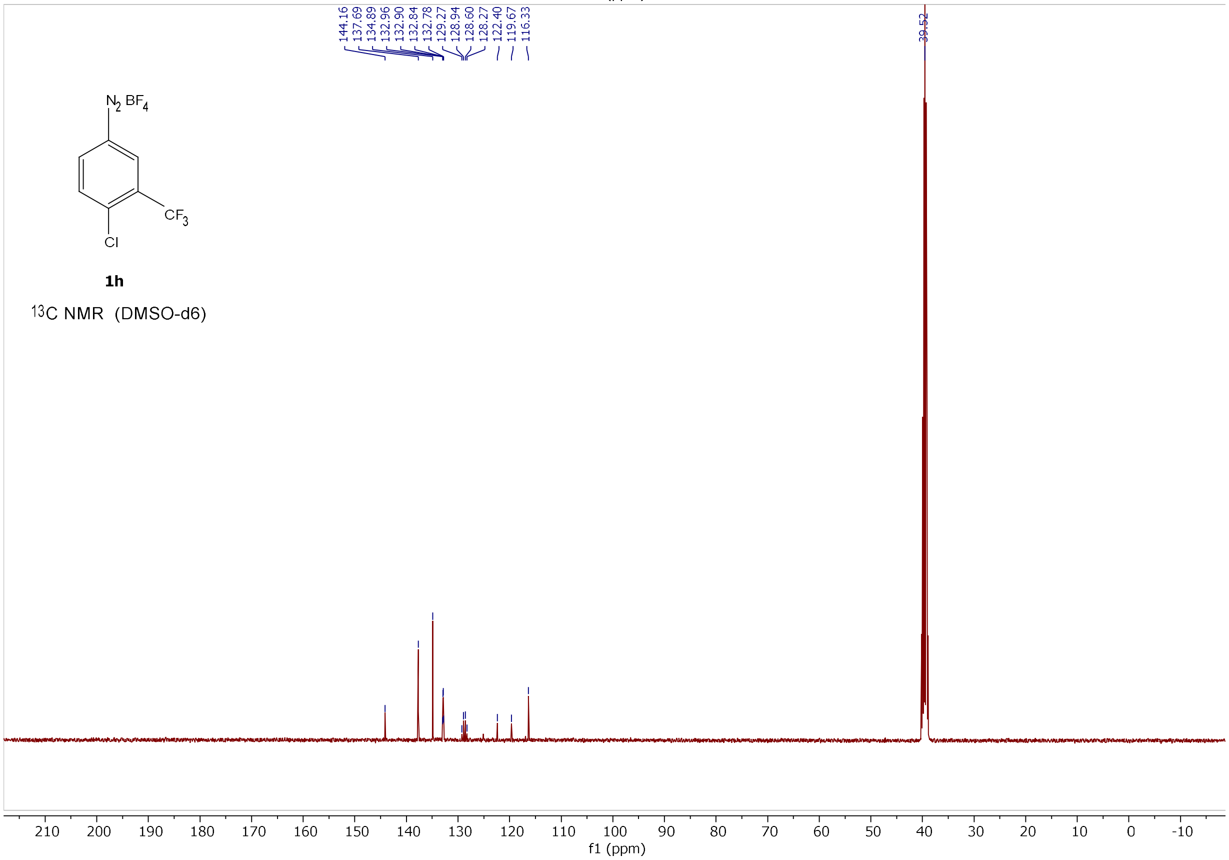
144.16
137.69
134.89
132.96
132.90
132.84
132.78
129.27
128.94
128.60
128.27
122.40
119.67
116.33
39.52
$N_2$ $BF_4$
$CF_3$
Cl
1h
13C NMR (DMSO-d6)
210 200 190 180 170 160 150 140 130 120 110 100 90 80 70 60 50 40 30 20 10 0 -10
f1 (ppm)

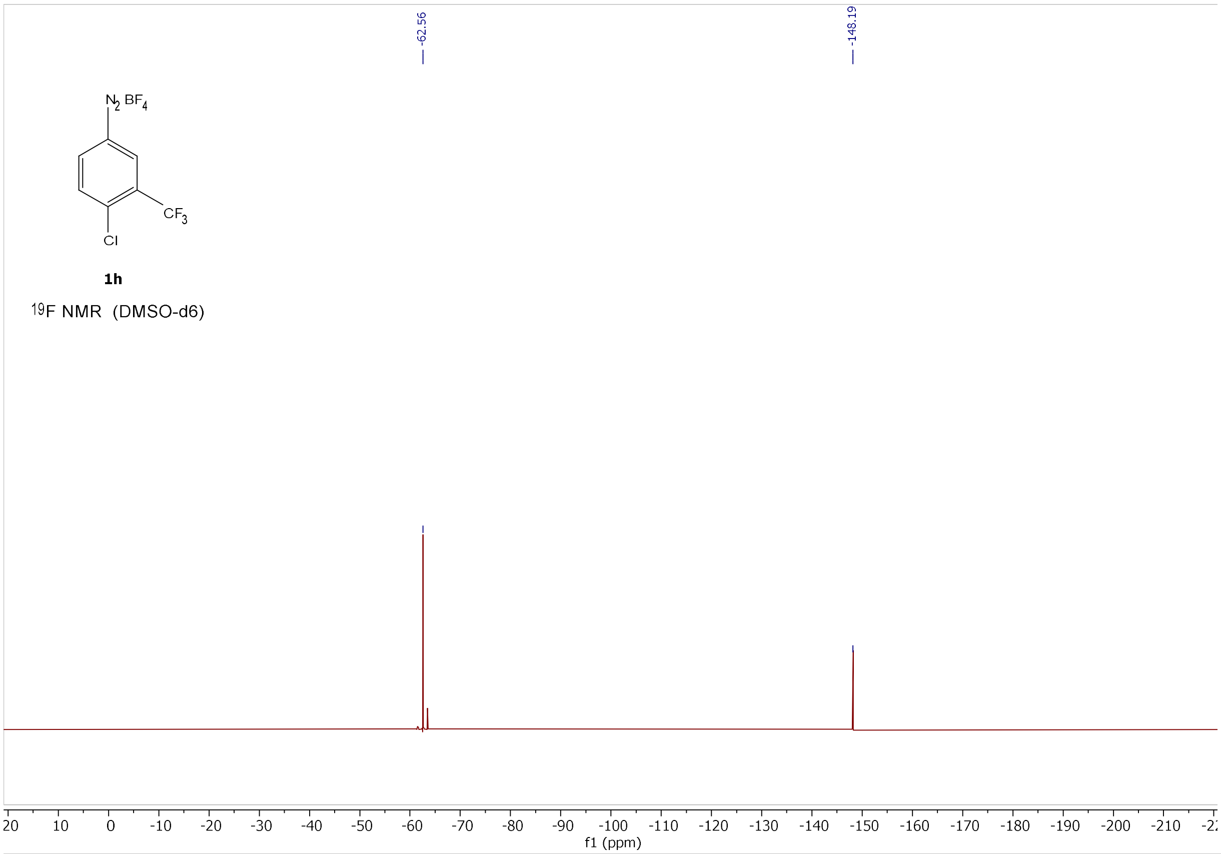
-62.56
-148.19
N2 BF4
CF3
Cl
1h
19F NMR (DMSO-d6)
20
10
0
-10
-20
-30
-40
-50
-60
-70
-80
-90
-100
-110
-120
-130
-140
-150
-160
-170
-180
-190
-200
-210
f1 (ppm)

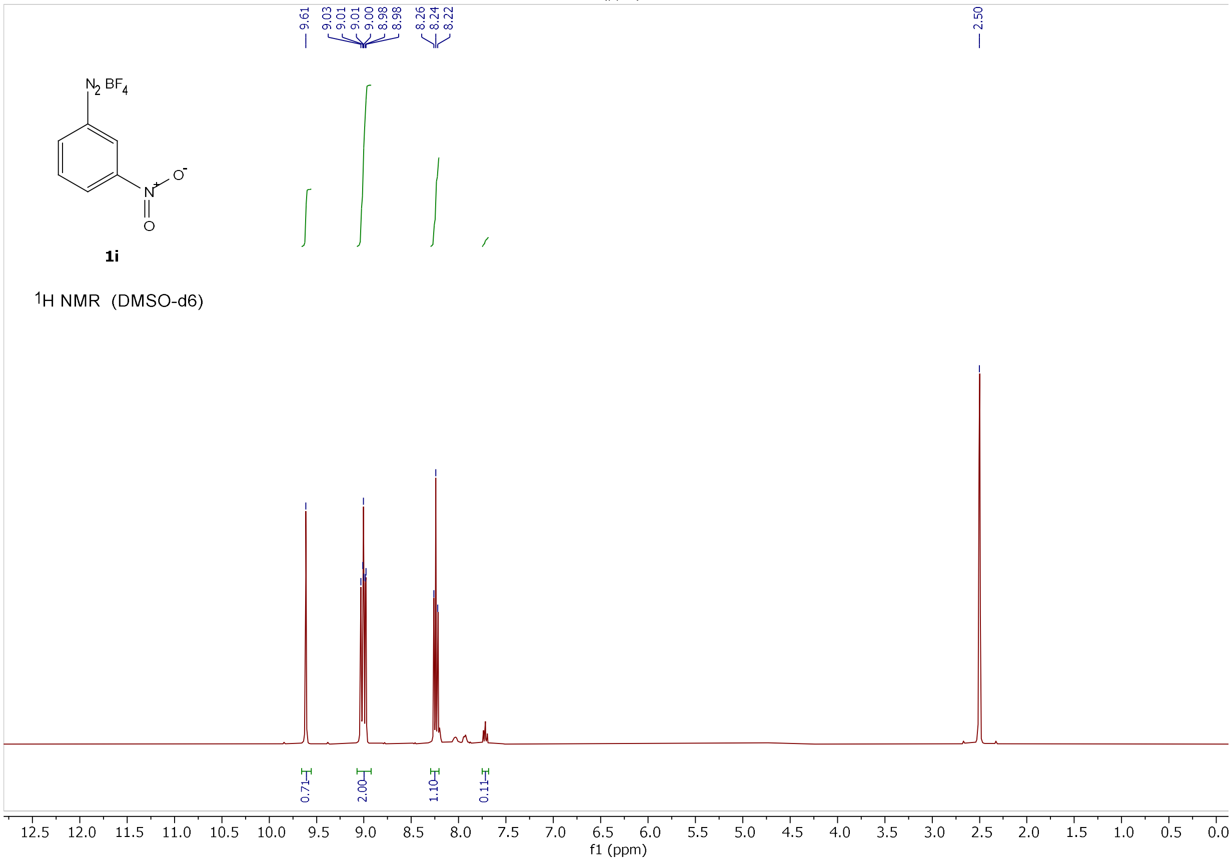
9.61
9.03
9.01
9.01
9.00
8.98
8.98
8.26
8.24
8.22
2.50
N2 BF4
N+
O-
O
1i
1H NMR (DMSO-d6)
0.71
2.00
1.10
0.11
12.5
12.0
11.5
11.0
10.5
10.0
9.5
9.0
8.5
8.0
7.5
7.0
6.5
6.0
5.5
5.0
4.5
4.0
3.5
3.0
2.5
2.0
1.5
1.0
0.5
0.0
f1 (ppm)

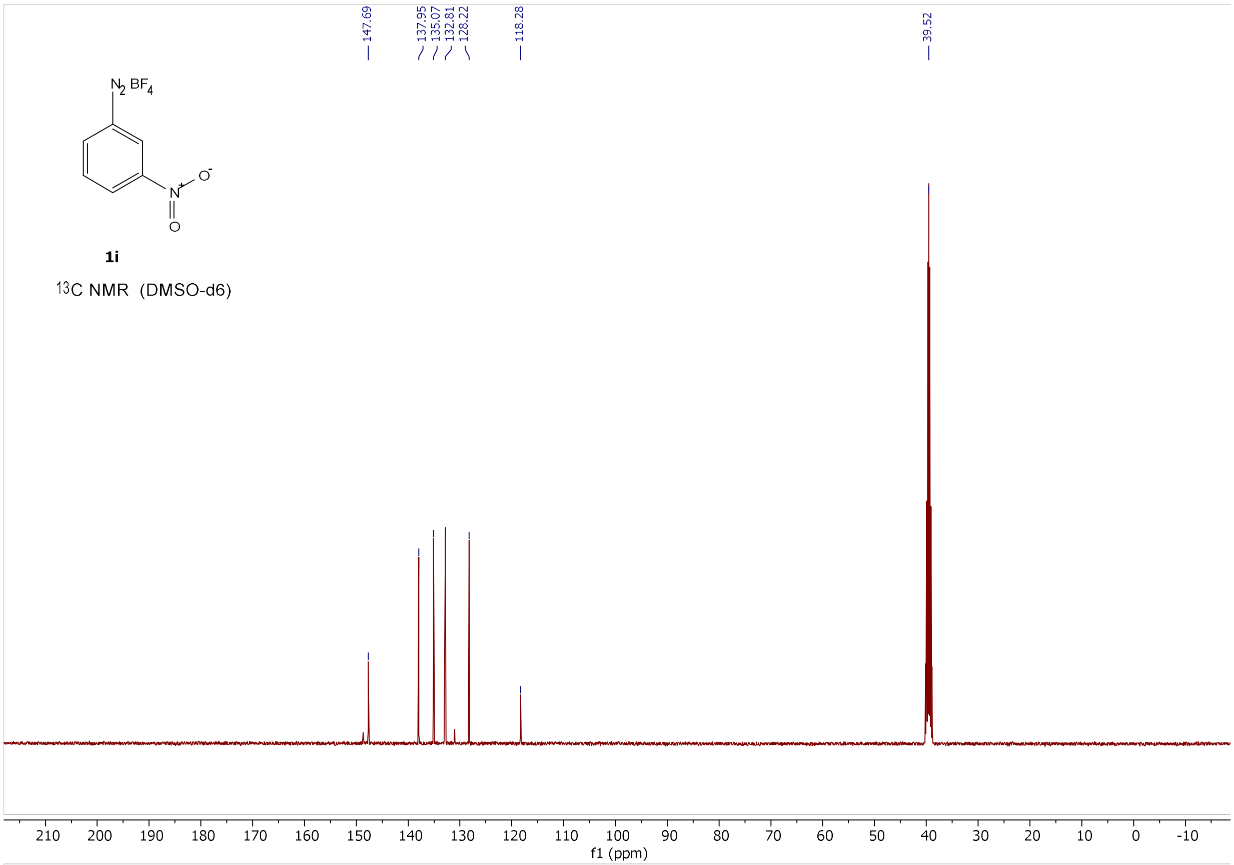
147.69
137.95
135.07
132.81
128.22
118.28
39.52
N2 BF4
1i
13C NMR (DMSO-d6)
f1 (ppm)

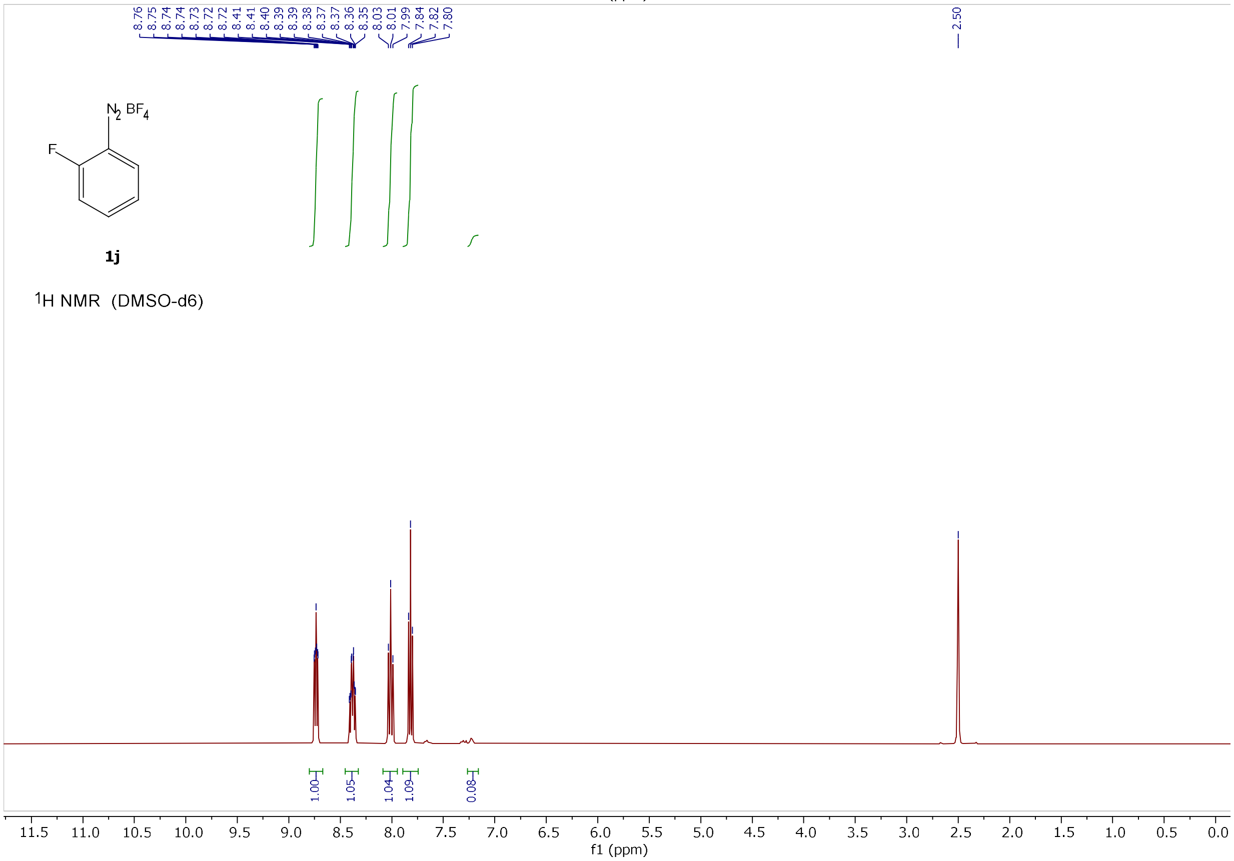
8.76
8.75
8.74
8.74
8.73
8.72
8.72
8.41
8.40
8.39
8.39
8.38
8.37
8.37
8.36
8.35
8.03
8.01
7.99
7.84
7.82
7.80
2.50
N2 BF4
F
1j
1H NMR (DMSO-d6)
1.00
1.05
1.04
1.09
0.08
f1 (ppm)

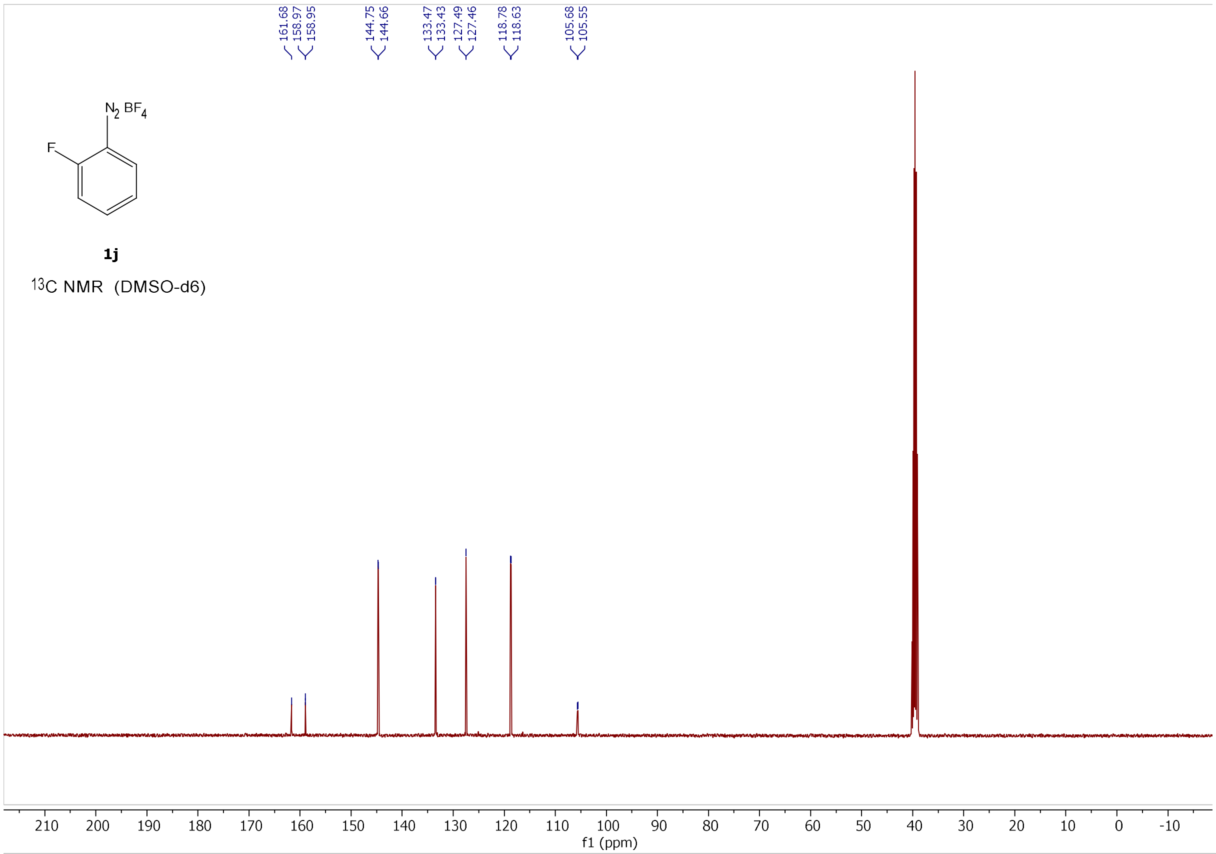


**1j**

$^{13}$C NMR (DMSO-d6)

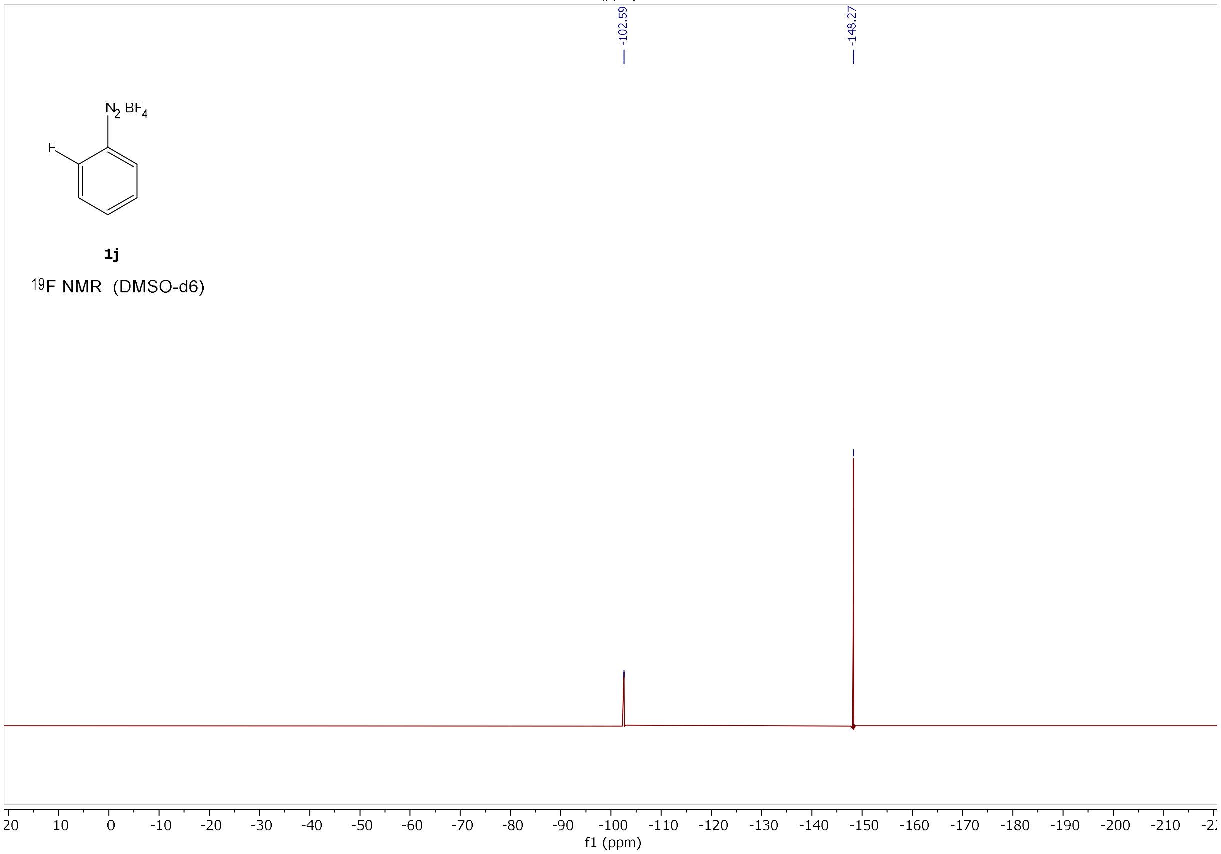


**1j**

$^{19}$F NMR (DMSO-d6)

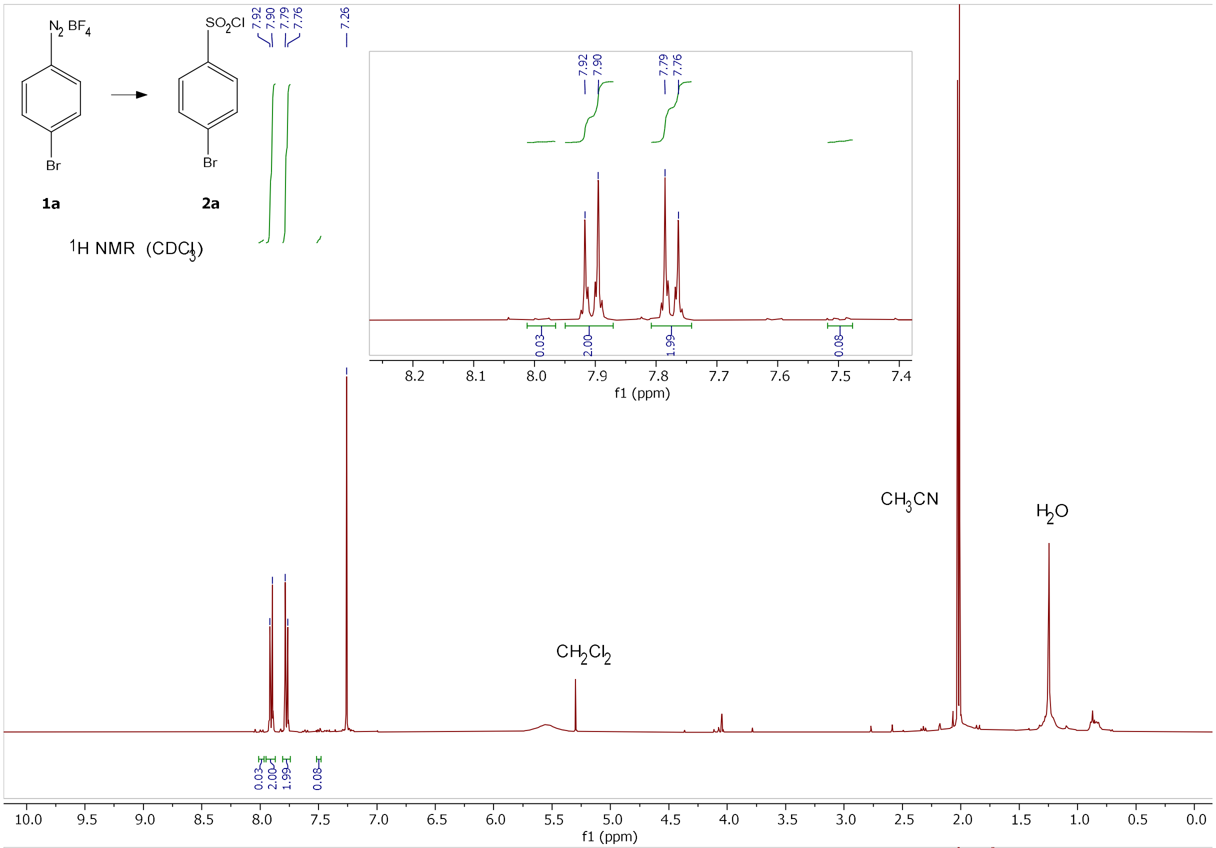
N2 BF4
SO2Cl
Br
Br
1a
2a
1H NMR (CDCl3)
7.92
7.90
7.79
7.76
7.26
CH3CN
H2O
CH2Cl2
0.03
2.00
1.99
0.08
f1 (ppm)

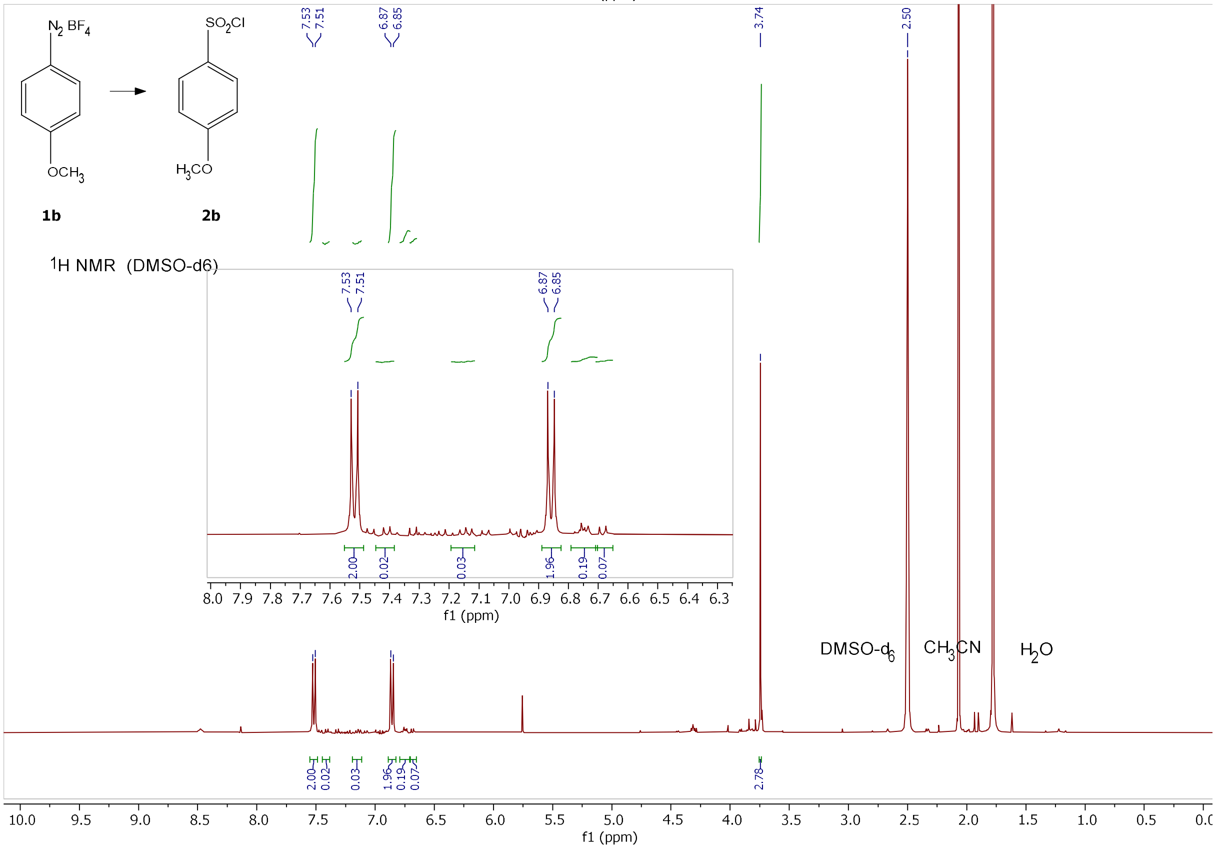
N2 BF4
SO2Cl
OCH3
H3CO
1b
2b
1H NMR (DMSO-d6)
7.53
7.51
6.87
6.85
3.74
2.50
DMSO-d6
CH3CN
H2O
2.00
0.02
0.03
1.96
0.19
0.07
2.78
f1 (ppm)

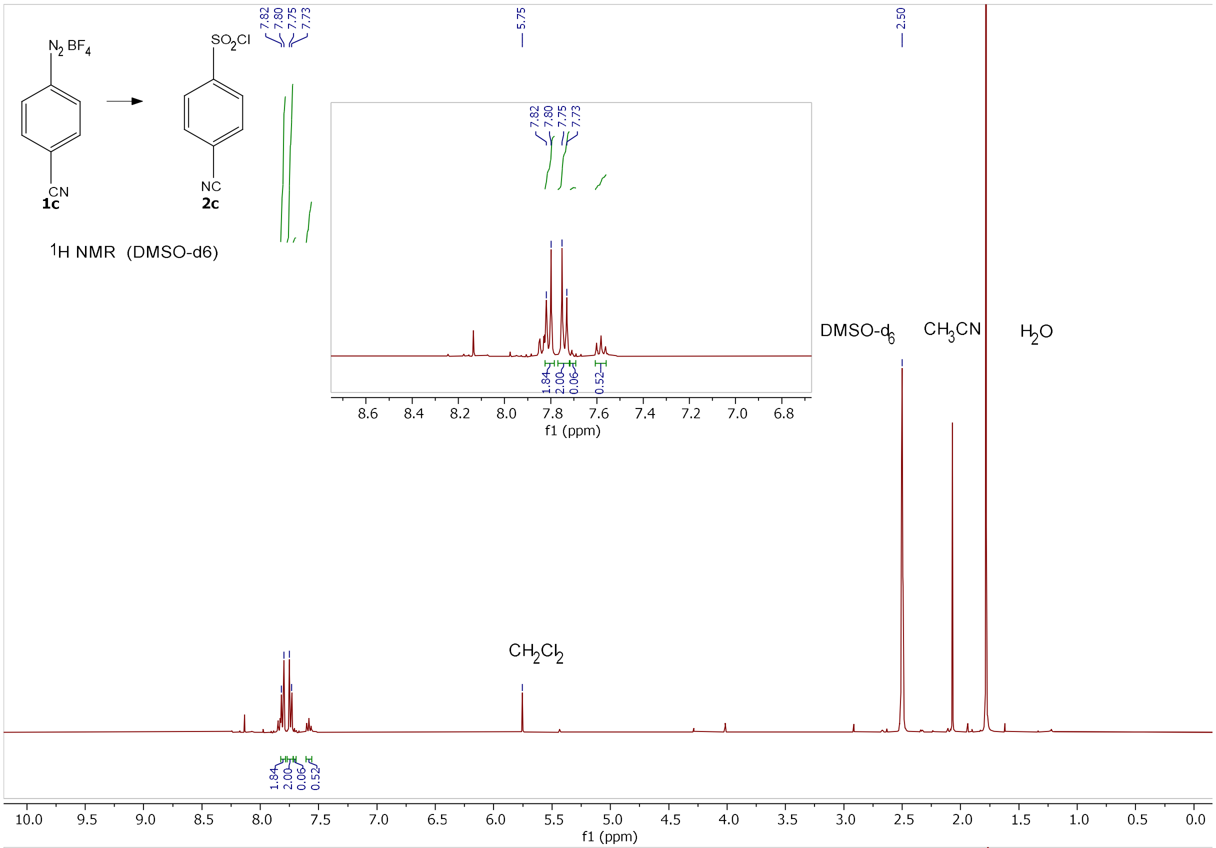
1c
2c
$^1$H NMR (DMSO-d6)
DMSO-d6
CH3CN
H2O
CH2Cl2
f1 (ppm)

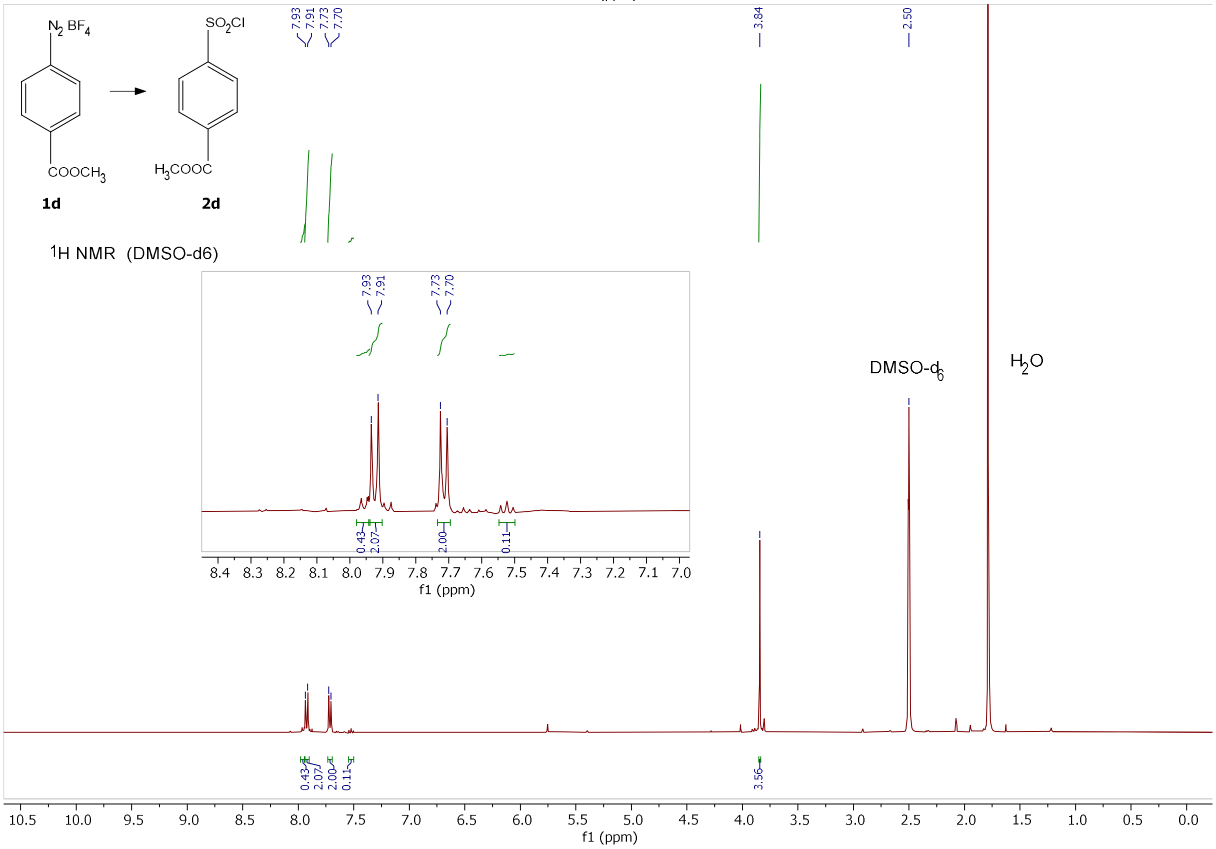
1d
2d
$^1$H NMR (DMSO-d6)
DMSO-d6
H2O
f1 (ppm)

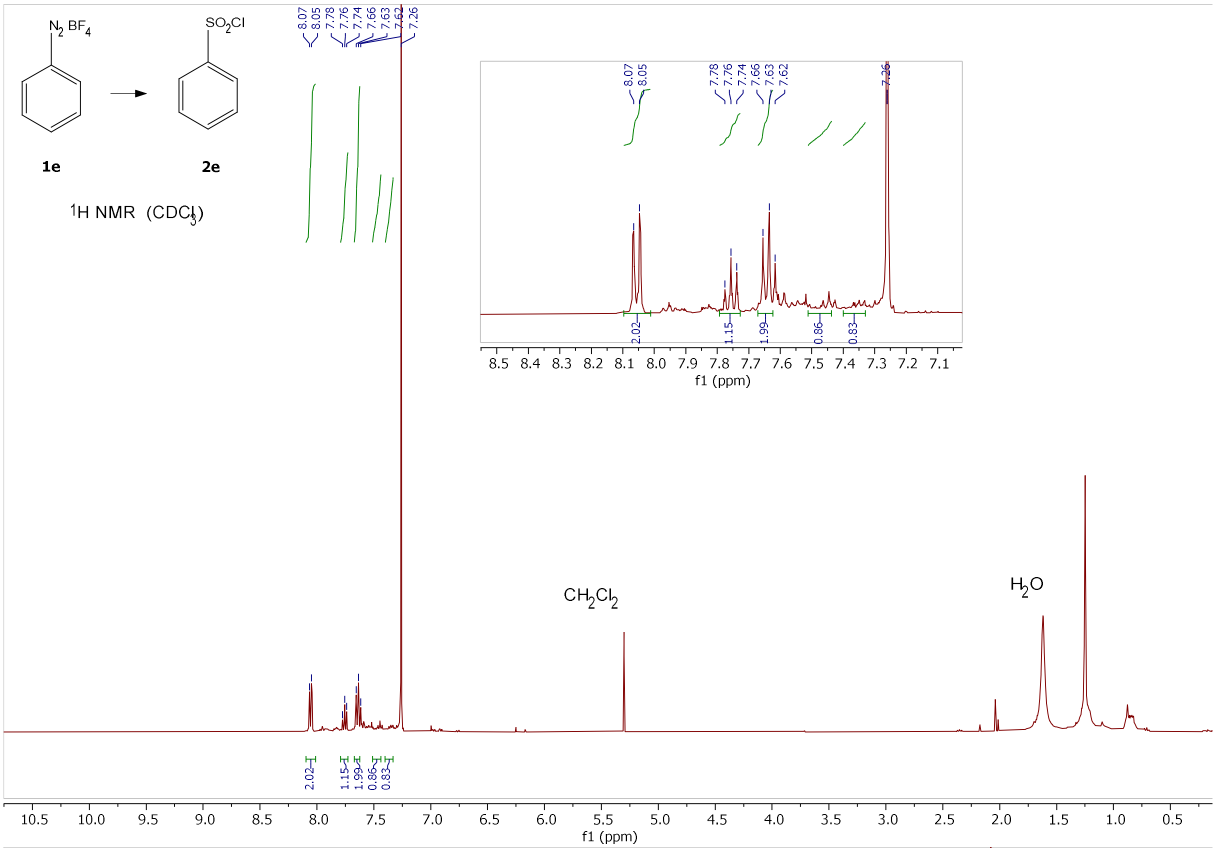

N2 BF4
SO2Cl
1e
2e
1H NMR (CDCl3)
8.07 8.05 7.78 7.76 7.74 7.66 7.63 7.62 7.26
2.02 1.15 1.99 0.86 0.83
CH2Cl2
H2O
f1 (ppm)


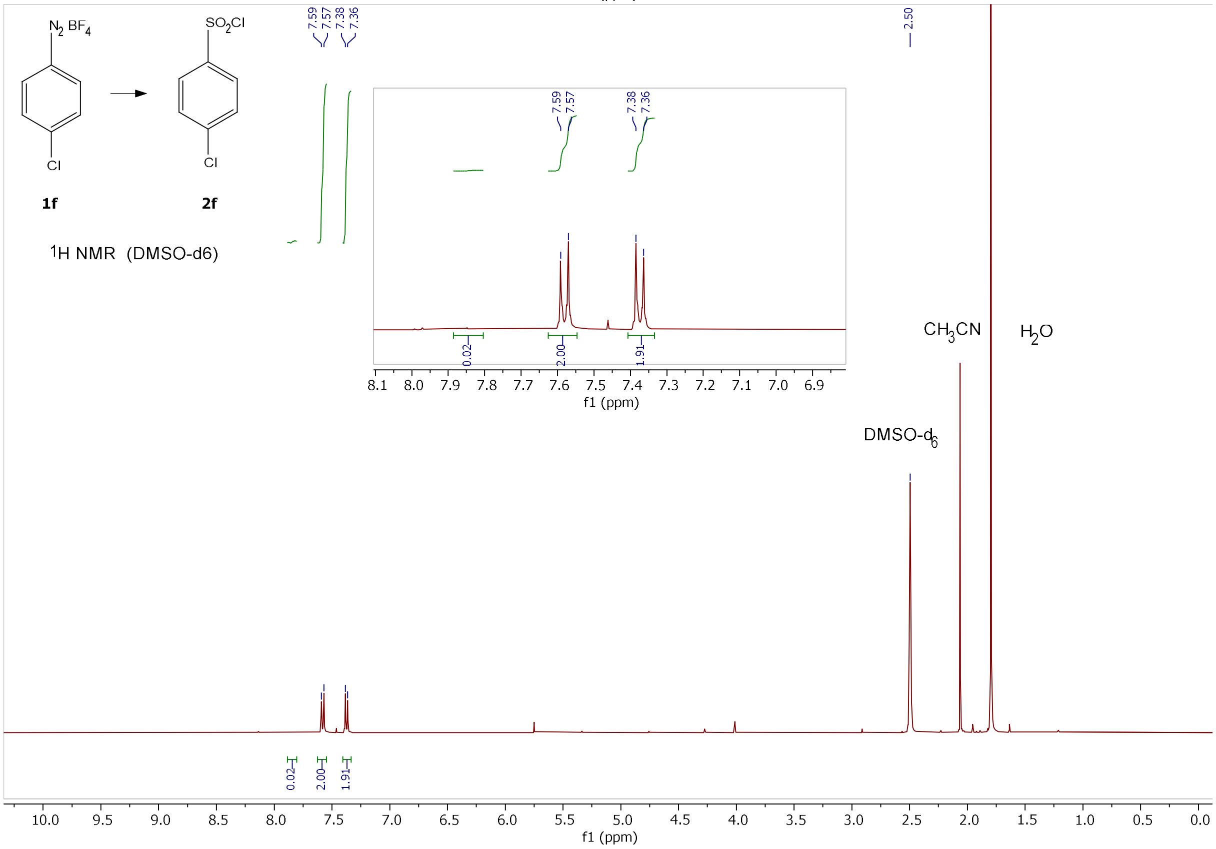

N2 BF4
SO2Cl
Cl
Cl
1f
2f
1H NMR (DMSO-d6)
7.59 7.57 7.38 7.36
2.50
0.02 2.00 1.91
CH3CN
H2O
DMSO-d6
f1 (ppm)

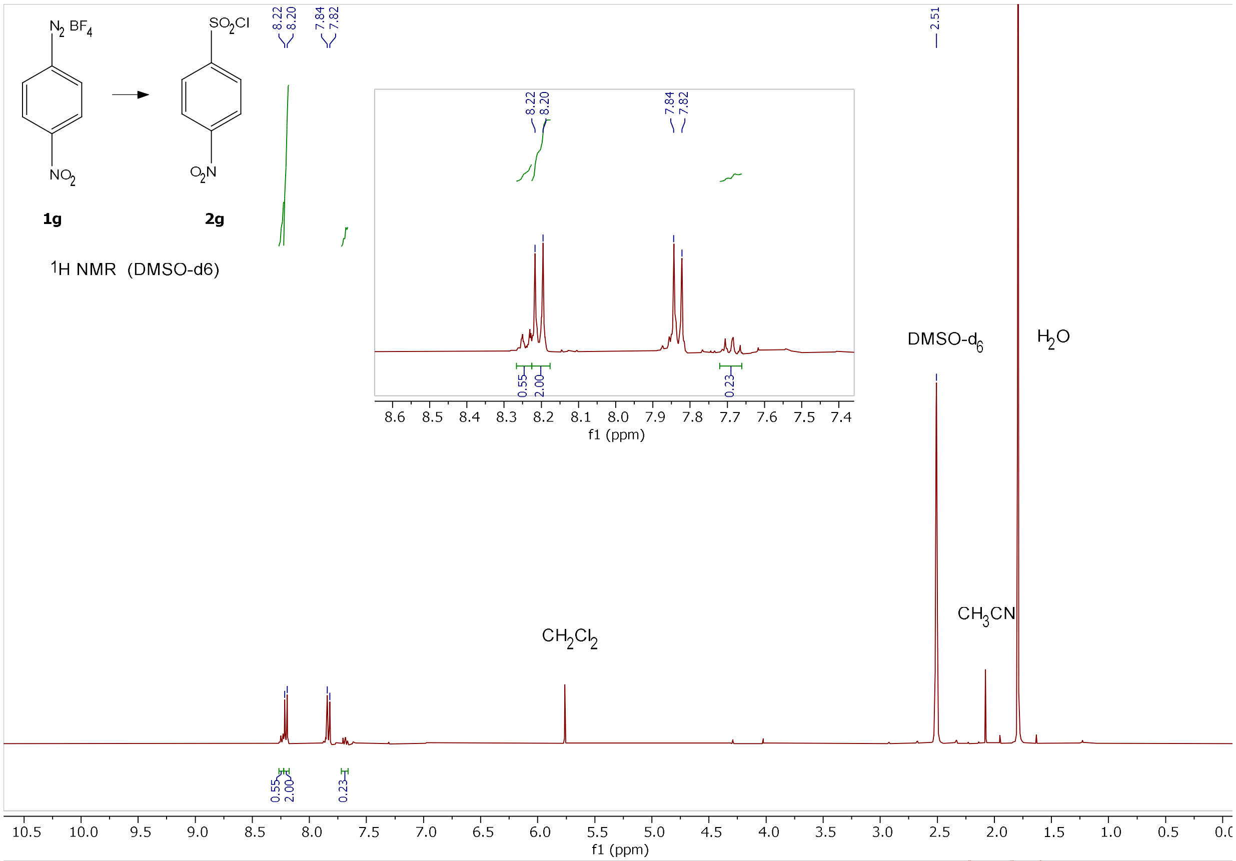

N2 BF4
SO2Cl
NO2
O2N
1g
2g
1H NMR (DMSO-d6)
8.22
8.20
7.84
7.82
2.51
DMSO-d6
H2O
CH3CN
CH2Cl2
0.55
2.00
0.23
f1 (ppm)


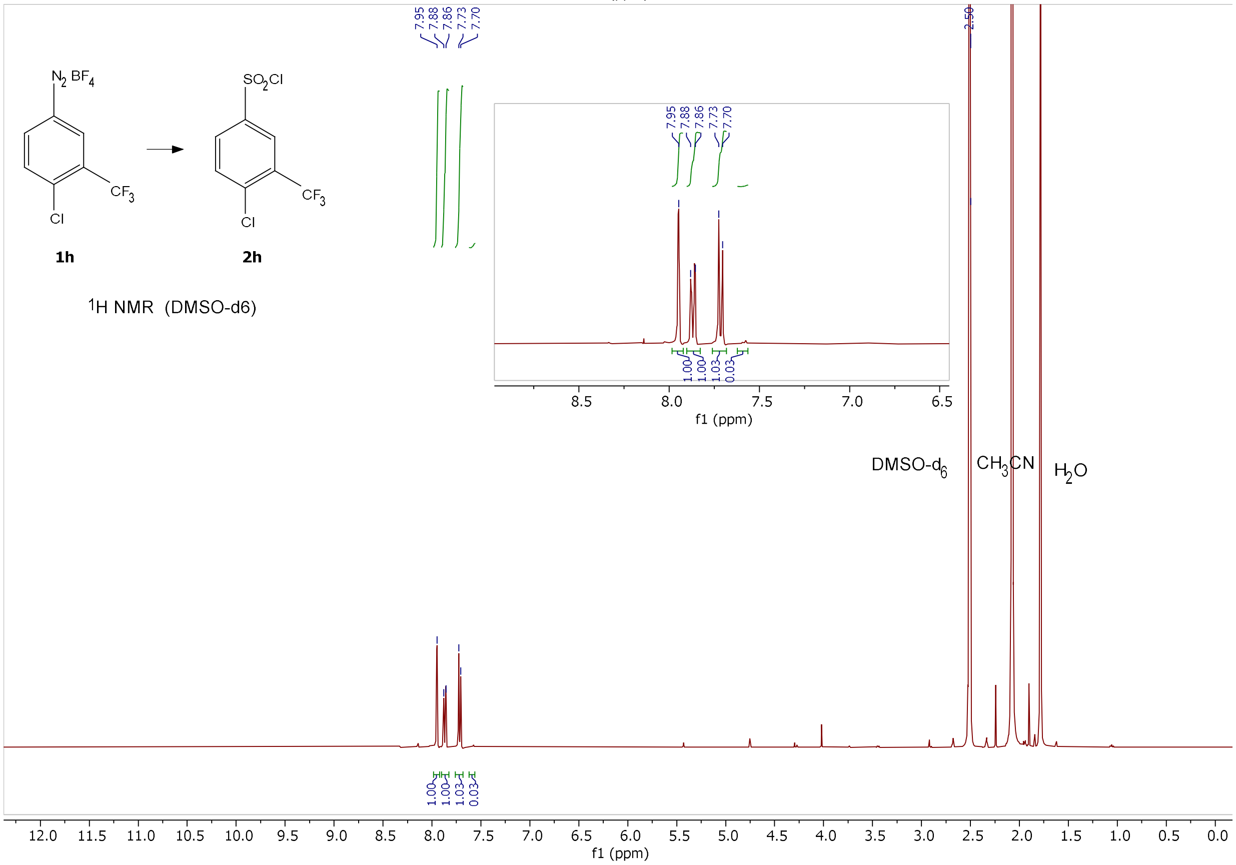

N2 BF4
SO2Cl
CF3
Cl
1h
2h
1H NMR (DMSO-d6)
7.95
7.88
7.86
7.73
7.70
2.50
DMSO-d6
CH3CN
H2O
1.00
1.00
1.03
0.03
f1 (ppm)

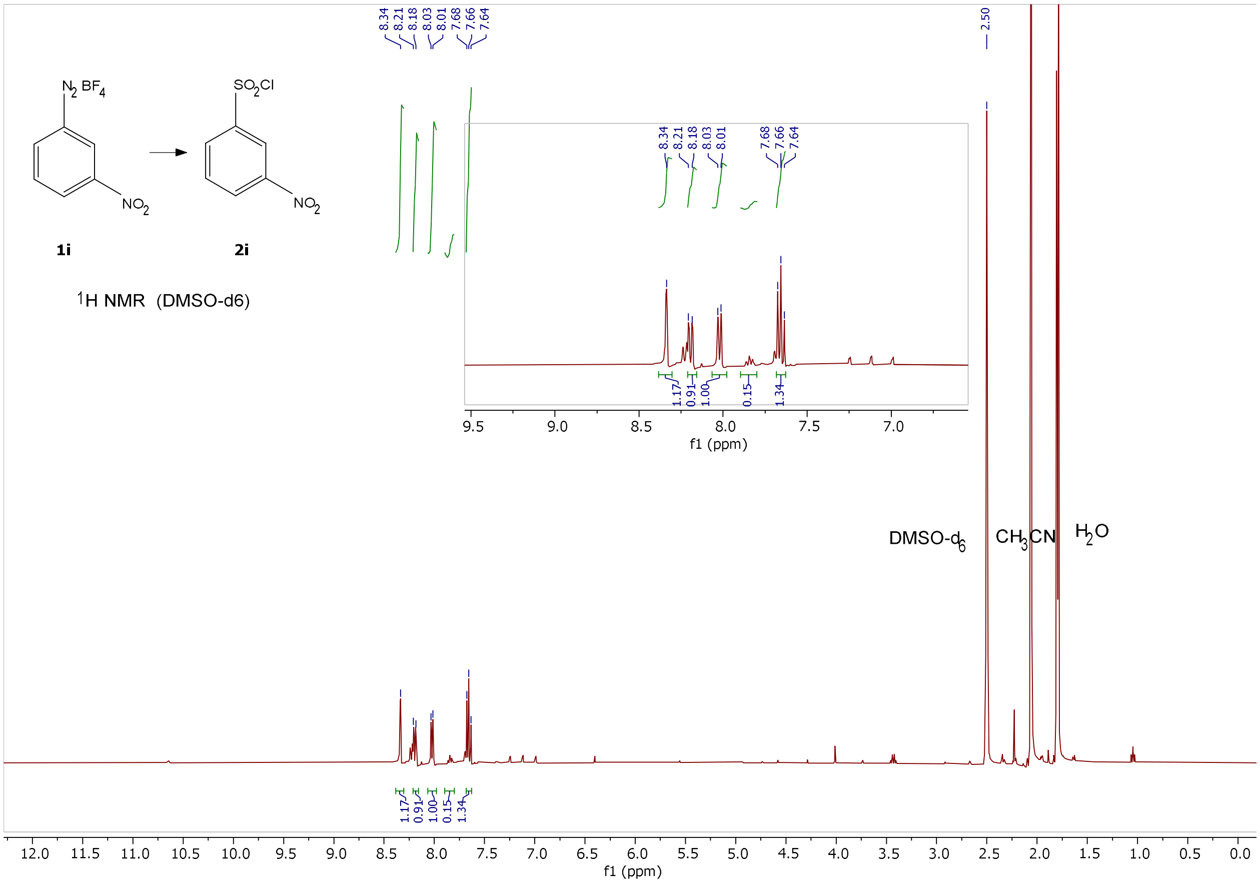

N2 BF4
SO2Cl
NO2
NO2
1i
2i
1H NMR (DMSO-d6)
8.34
8.21
8.18
8.03
8.01
7.68
7.66
7.64
2.50
1.17
0.91
1.00
0.15
1.34
DMSO-d6
CH3CN
H2O
f1 (ppm)

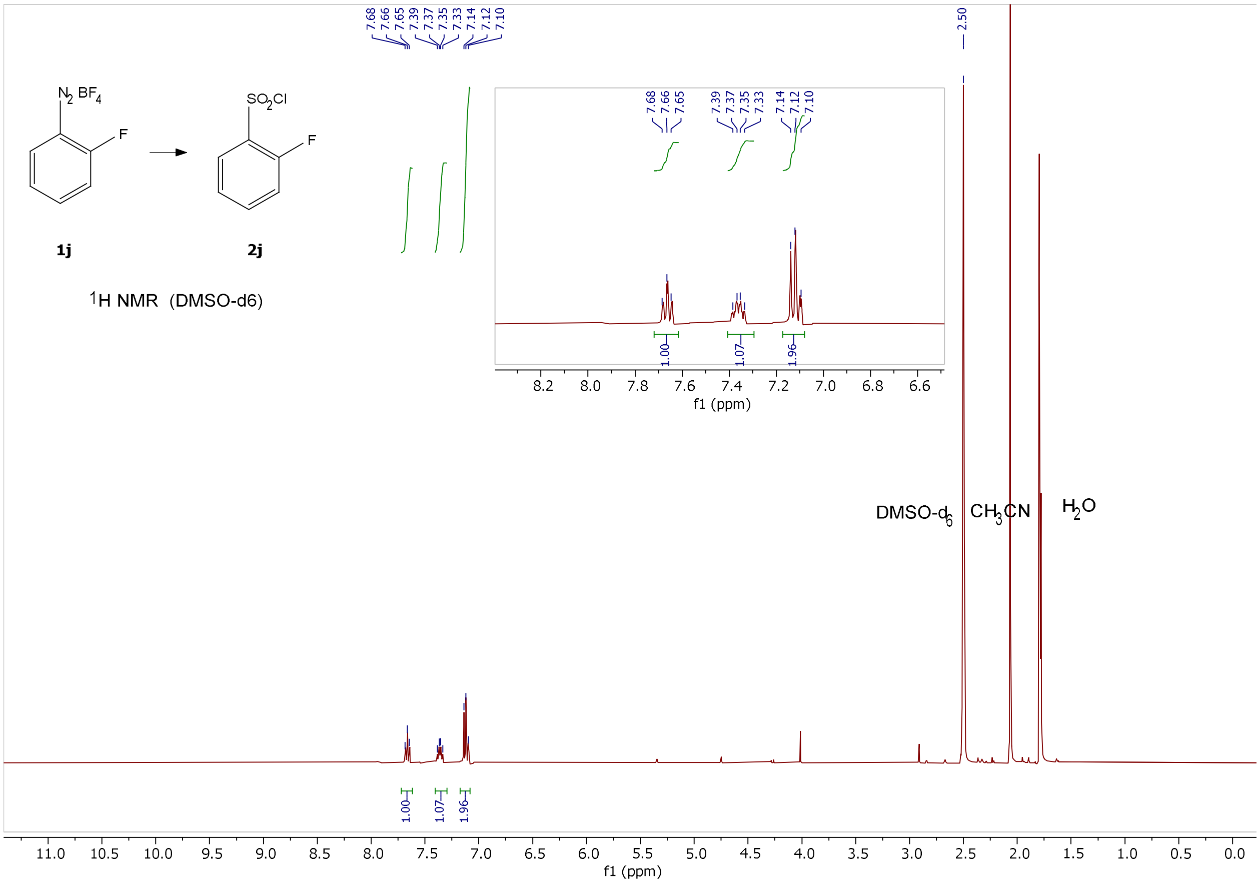


# References


1. Savateev, A.; Dontsova, D.; Kurpil, B.; Antonietti, M., Highly crystalline poly(heptazine imides) by mechanochemical synthesis for photooxidation of various organic substrates using an intriguing electron acceptor – Elemental sulfur. *J Catal* **2017,** *350*, 203-211.

2. Kurpil, B.; Otte, K.; Mishchenko, A.; Lamagni, P.; Lipiński, W.; Lock, N.; Antonietti, M.; Savateev, A., Carbon nitride photocatalyzes regioselective aminium radical addition to the carbonyl bond and yields N-fused pyrroles. *Nature Communications* **2019,** *10* (1), 945.

3. Ghosh, I.; Khamrai, J.; Savateev, A.; Shlapakov, N.; Antonietti, M.; König, B., Organic semiconductor photocatalyst can bifunctionalize arenes and heteroarenes. *Science* **2019,** *365* (6451), 360-366.

4. Chen, Z.; Savateev, A.; Pronkin, S.; Papaefthimiou, V.; Wolff, C.; Willinger, M. G.; Willinger, E.; Neher, D.; Antonietti, M.; Dontsova, D., "The Easier the Better" Preparation of Efficient Photocatalysts-Metastable Poly(heptazine imide) Salts. *Advanced Materials* **2017,** *29* (32).

5. Qian, L.-W.; Sun, M.; Dong, J.; Xu, Q.; Zhou, Y.; Yin, S.-F., Palladium-Catalyzed Desulfitative Cross-Coupling of Arylsulfonyl Hydrazides with Terminal Alkynes: A General Approach toward Functionalized Internal Alkynes. *The Journal of Organic Chemistry* **2017,** *82* (13), 6764-6769.

6. Hari, D. P.; Schroll, P.; Konig, B., Metal-free, visible-light-mediated direct C-H arylation of heteroarenes with aryl diazonium salts. *J Am Chem Soc* **2012,** *134* (6), 2958-61.

7. Hamada, T.; Yonemitsu, O., An Improved Synthesis of Arylsulfonyl Chlorides from Aryl Halides. *Synthesis* **1986,** *1986* (10), 852-854.
8. Attia, M. I.; Timmermann, M.; Högger, P.; Herdeis, C., Design, Synthesis and Biological Activity of Azasugar-Based CD163 Ectodomain Shedding Inhibitors. *Eur. J. Org. Chem.* **2007,** *2007* (22), 3669-3675.
9. Silva-Cuevas, C.; Perez-Arrieta, C.; Polindara-García, L. A.; Lujan-Montelongo, J. A., Sulfonyl halide synthesis by thiol oxyhalogenation using NBS/NCS – iPrOH. *Tetrahedron Lett.* **2017,** *58* (23), 2244-2247.
